# Development of the Sinus Headache Screener to identify patients with non-rhinogenic facial pain compared with chronic rhinosinusitis in rhinology clinics

**DOI:** 10.1186/s41687-025-00956-4

**Published:** 2025-11-06

**Authors:** Theresa Coles, Laura S. Mkumba, Lauren Wright, Frederick A. Godley, Timothy A. Collins, David W. Jang

**Affiliations:** 1 Department of Population Health Sciences, Center for Health Measurement, Duke University School of Medicine, Durham, NC USA; 2 Association of Migraine Disorders, North Kingstown, RI USA; 3 Department of Neurology, Duke University School of Medicine, Durham, NC USA; 4 Department of Head and Neck Surgery & Communications Sciences, Duke University School of Medicine, Durham, NC USA

**Keywords:** Screening, Case finding, Questionnaire development, Patient-reported outcome measure, Cognitive interviews

## Abstract

**Purpose:**

To develop a patient-reported screening tool, the Sinus Headache Screener (SHS), to differentiate non-rhinogenic facial pain (NRFP) from chronic rhinosinusitis (CRS) using qualitative research methods.

**Methods:**

We conducted semi-structured interviews with 26 English-speaking adults (15 NRFP, 11 CRS). Interviews included concept elicitation and cognitive interviewing techniques. Content analysis was used to analyze transcripts, and items were iteratively refined over three rounds of interviews.

**Results:**

The final list of potential SHS items consisted of 89 items across 8 sections, including symptoms, episode characteristics, triggers, and treatments.

**Conclusions:**

This study represents the first step in developing a screening questionnaire to identify patients with NRFP in clinical settings. The item list provides a foundation for future quantitative studies to refine the questionnaire, potentially leading to more appropriate diagnosis and treatment of patients presenting with facial pain or pressure.

**Supplementary Information:**

The online version contains supplementary material available at 10.1186/s41687-025-00956-4.

## Introduction

Sinus symptoms are among the most common complaints in primary care. Many patients present with chronic or recurrent pain or pressure around the sinuses, often leading to a diagnosis of chronic rhinosinusitis (CRS). However, evidence suggests that many of these patients actually have non-rhinogenic facial pain or pressure (NRFP) with a migraine etiology [1, 2]. Despite this growing body of literature, patients with NRFP are frequently misdiagnosed with CRS, even when there is no objective evidence of CRS. This misdiagnosis leads to ineffective utilization of healthcare resources, including unnecessary antibiotics, physician visits, radiologic studies, and surgeries [3, 4].

The core issue is the lack of diagnostic support for clinicians to guide patients to the most appropriate initial treatment, thus minimizing unnecessary healthcare resource utilization. While symptom-based tools for CRS are available, these tools typically have low specificity and are better suited for assessing response to therapy [5]. Existing screening questionnaires for migraines, developed for patients meeting International Headache Society criteria for classic or common migraine, are likely not fit-for-purpose for patients presenting with NRFP and concomitant sinus-type symptoms, as they focus solely on headache-specific symptoms [6].

Considering NRFP as a migraine variant condition suggests that integrating concepts from both CRS and migraine could be a strong foundation for differentiating these diagnoses. One approach to triage patients with sinus and nasal complaints is to introduce a screening questionnaire based on comorbid symptoms and exacerbating factors specific to NRFP. Several studies suggest that this approach is feasible [1, 2, 7–9].

The first step in developing a fit-for-purpose screening questionnaire for use in clinical care is to conduct qualitative concept elicitation and cognitive debriefing interviews [10, 11]. Concept elicitation ensures that the questionnaire content is relevant to patients and comprehensively captures patient-reported symptoms and experiences that should be considered for inclusion in the questionnaire. Concept elicitation methods also provide an opportunity to examine themes in patient experiences that may be key drivers differentiating NRFP from CRS. Cognitive interviewing, a technique that aims to describe how people get to their answer when presented with the wording of a question, assess patients’ comprehension of questionnaire items, response options, and instructions to ensure content validity, appropriate response choices, and recall periods. Without these qualitative steps, screening questionnaires are at risk for being misunderstood by patients, thus integrating unnecessary error into the detection of potential diagnoses.

The purpose of this study is to develop a draft item list (i.e., list of survey questions) for the Sinus Headache Screener (SHS) using concept elicitation and cognitive interviewing techniques. The long-term goal of this patient-oriented screening questionnaire is to support clinicians in the early proper diagnosis and treatment of patients, leading to more rapid and appropriate care, reduced costs, and less patient suffering and disability.

## Methods

Within an interpretative research paradigm [12], one-on-one qualitative interviews were conducted with English-speaking adults ≥ 18 years of age presenting with a diagnosis of CRS or suspected NRFP at the [BLINDED] University Medical Center rhinology clinics. Exclusion criteria included history of genetic or autoimmune disease affecting the sinuses (e.g., cystic fibrosis, sarcoidosis) and significant cognitive or memory impairment that would render it difficult for the participant to interact in an interview. Patients with other known causes of facial pain (e.g., dental infection, trauma, neoplasm, temporomandibular disorders) were excluded.

The qualitative study team included two clinicians, one clinical research coordinator (LM), one clinical research specialist (LW), and a PhD-level health researcher (TC). The research team was joined by an advisory panel of experts, including a patient with NRFP, five physicians with expertise in migraine, otolaryngology, and neurology, and one dentist (DDS) with expertise in facial pain. Advisory panel members met with the research team before the study began to review and provide input on draft items and provide insight on existing and new items between rounds.

### Sample size determination

Our sample size was informed by previous research [13] showing that little new information (saturation) was learned after 12 interviews within a homogeneous sample. Using purposive sampling [14], we sought to enroll 12 patients with a diagnosis of CRS and 18 patients with a diagnosis of NRFP. The NRFP group was intentionally larger because it includes patients with more diverse experiences from migraine and non-migraine etiology. During data collection, participant characteristics were tracked in an effort to achieve a balanced sample related to background characteristics including age (18–59; ≥60) [age of individuals may influence the impacts on activities], race (Black or African American, White, or other)ethnicity, and gender [to ensure the sample represented diverse backgrounds], education [as a proxy for health literacy to test the SHS at different levels].

### Diagnostic classifications

Diagnostic criteria for NRFP included chronic or episodic pain or pressure of the midface for the last 3 months with normal findings on nasal endoscopy at initial presentation and no evidence of abnormal sinus findings on imaging (CT or MRI) obtained when symptomatic in the last 12 months prior to presentation. Patients who had prior surgery with complete visualization of all sinuses were exempt from imaging. The midface includes the region overlying the maxillary, ethmoid, and frontal sinuses. Normal imaging findings included isolated mucus retention cysts 2 cm or less in size and minimal non-circumferential mucosal thickening (3 mm or less). Normal endoscopy was defined as the absence of edema or purulent drainage at the middle meatus or sphenoethmoid recess. Diagnostic criteria for CRS were based on American Academy of Otolaryngology Clinical Practice Guidelines [15].

### Participant recruitment

Potential participants were identified by rhinology clinicians in the outpatient clinics of [BLINDED] University Medical Center. Study team members then reached out to potentially eligible patients via email or phone to share information about the study and obtain verbal consent. A brief verbal screening questionnaire was administered to confirm eligibility (Appendix 1) and ensure a balance of patient characteristics (see sample size determination). Study team members reviewed the verbal consent form with interested eligible patients. Following affirmative verbal consent, participants were scheduled for a future interview via their preferred method of either web video conferencing (Zoom) or phone call. Participants were also sent a copy of the study information sheet which described the study’s procedures, noted that participation was completely voluntary, and advised participants of their right to withdraw their participation at any time without influence to their clinical care.

### Interviewing method

**Fig. 1:**
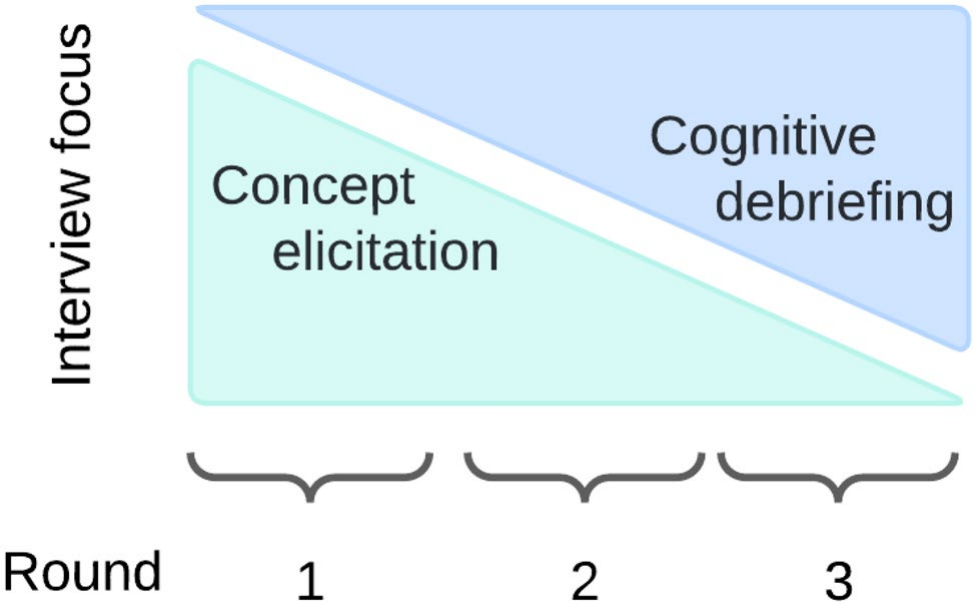
Focus of interviews

Semi-structured interviews were conducted in three rounds to enable additions and improvements to the item list between rounds and subsequent testing for those changes in future rounds. With each subsequent round, we transitioned from more time spent in the interview on open information about patients’ experiences (concept elicitation) to more time spent on cognitive interviewing (Fig. 1). This novel study design allowed content validity evidence to be collected (supporting the development or modification of items/concepts) and accelerated the development of the SHS items. The study team and advisory panel developed and refined the initial interview guide.

### Interview content

**Fig. 2:**
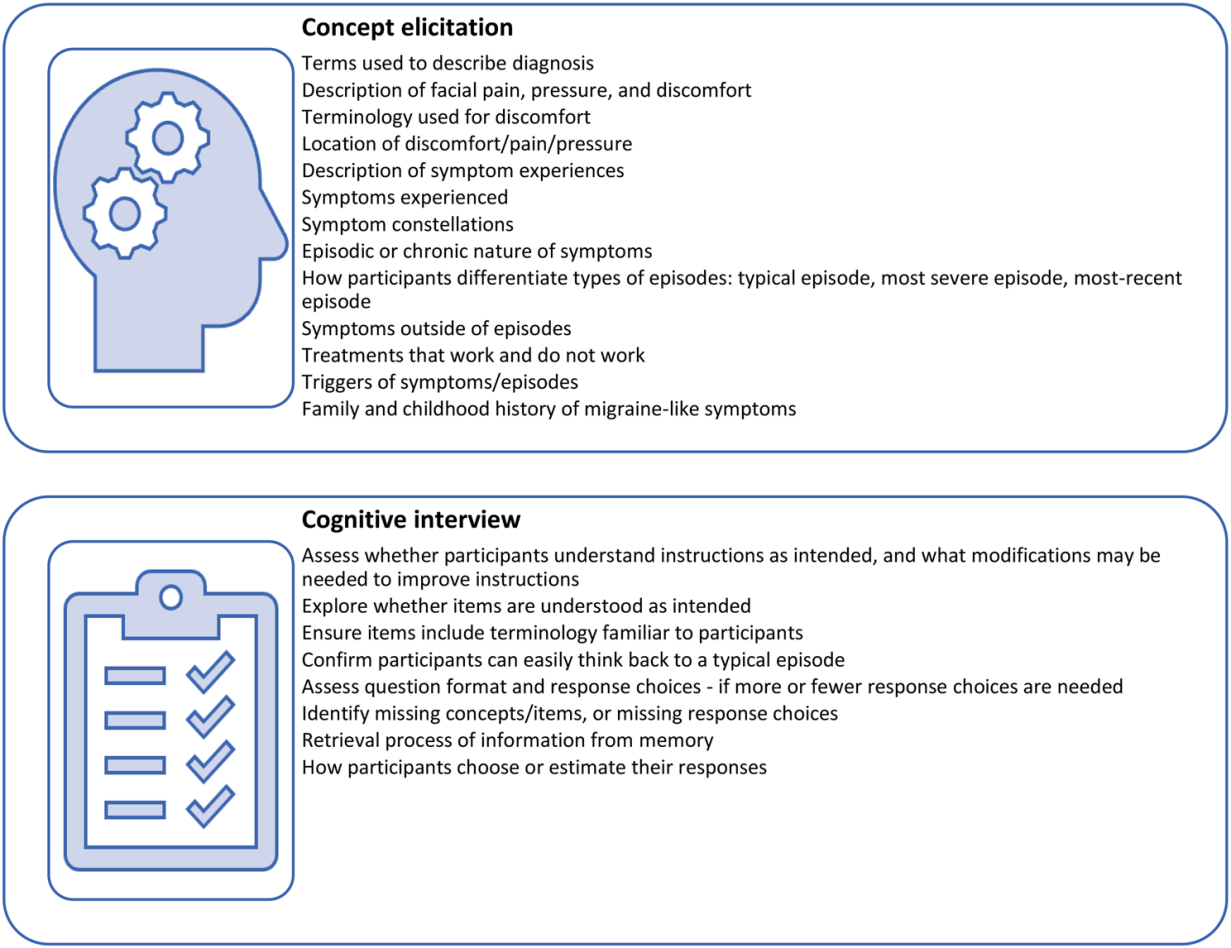
Concepts examined during qualitative interviews

An overview of topics covered in the interviews is presented in Fig. 2. Interview guides for each round are presented in Appendix 2.

Interviews were conducted via phone or web (with the camera on) from February through July of 2022 by two interviewers (LM, LW) who were trained in concept elicitation and cognitive interviewing methods. They were audio-recorded and transcribed verbatim for analysis. Two interviews were not transcribed due to poor audio quality. Interviewers completed debriefing forms after each interview. Debriefing forms are formal, structured interviewer notes used to document key findings, interviewer impressions, and nonverbal ques. Participants received remuneration of $25. The study was approved as an exempt protocol by the [BLINDED] University Institutional Review Board (Pro00107312) on November 24, 2020.

### Analytic methods

Descriptive statistics were calculated to summarize characteristics of the study participants. Qualitative content analysis was used to analyze participant narratives and summarize evidence for content validity of the SHS items [16]. A codebook was developed iteratively among investigators (Appendix 3). The team used a hybrid top-down and bottom-up approach to code patient interview transcripts and debriefing forms if the transcript was unavailable. The hybrid approach provided structure for the concepts that were planned to be discussed a priori, as well as flexibility to identify and code developing themes. Coding was conducted in NVivo. The first transcript was coded as a team (TC, LM, LW) to ensure consistent use of codes. Coding agreement was assessed twice (one transcript from two different rounds) during the coding process to ensure consistency over time. During this process, two analysts (LM, LW) coded the same transcript. Coders compared code segments and agreement was tracked. Discrepancies were discussed and documented, the codebook was reviewed and revised, reasons for codebook changes were documented, and all previously coded transcripts were re-reviewed to apply coding changes.

Study team members (LM, LW) examined code output by diagnosis and developed short code summaries, focusing on the differences in experiences among individuals classified as having NRFP versus CRS. Debriefing forms were reviewed to provide context to the transcripts. The study team kept track of changes to items between rounds using an item tracking matrix (Appendix 4). Percentages were reported only when the analysis team could calculate a denominator based on the number of participants who were asked a question in the interviews.

Given that this is a qualitative study using purposive sampling, we caution readers about making inferences about the prevalence of phenomena observed beyond the sample.

## Results

The team attempted to contact 47 patients, and of these, interviewers were able to speak with 39. Of the 39, none were ineligible, 6 declined, 7 were lost to follow up, and 26 participated. Four out of the six patients who declined participation provided their reasons for refusal. The reasons were confidentiality concerns (*n* = 2), discomfort with associated risks (*n* = 1), poor health/difficulty speaking (*n* = 1). Table 1 presents characteristics of the 26 study participants. Fifteen were diagnosed with NRFP and 11 with CRS. Using debriefing forms and a redundancy tracking matrix (Appendix 4), it was determined that saturation was reached by 26 interviews. 80% of participants were female, and a quarter of the sample was Black/African American. The number of participants in each round was relatively even with 9 participants in round 1, 9 in round 2, and 8 in round 3 (Appendix 5).

**Table 1:** Participant characteristics by diagnosis and overall

Characteristics	NRFP (*n* = 15)	CRS (*n* = 11)	Overall (*n* = 26)
**Age** [mean (SD), median, min-max]	54.1 (14.4), 58.0, 25–81	59.6 (14.2), 63, 38–81	50.1 (13.6), 51.0, 25–68
**Gender**			
Women	14 (93.3%)	7 (63.6%)	21 (80.8%)
**Spanish/Hispanic/Latino origin**	0 (00.0%)	0 (00.0%)	0 (00.0%)
**Racial background (check all that apply)**			
White	11 (73.3%)	9 (81.8%)	20 (76.9%)
Black or African American	4 (26.7%)	2 (18.2%)	6 (23.1%)
**Education**			
Less than high school or high school graduate or equivalent (e.g., GED)	1 (6.7%)	1 (9.1%)	2 (7.7%)
Completed some college, associate’s degree, or college graduate	7 (46.7%)	4 (36.4%)	11 (42.3%)
Completed graduate school	7 (46.7%)	6 (54.5%)	13 (50.0%)
**Interview format**			
Phone	0 (00.0%)	1 (9.1%)	1 (3.8%)
Zoom	15 (100.0%)	10 (90.9%)	25 (96.2%)

### Part 1: Concept elicitation results

Results of the concept elicitation are described here, with illustrative quotes from participants in Table 2.

#### Terms used to describe diagnoses

Interviews found little recognition of the term “non-rhinogenic facial pain” or “NRFP,” but participants generally agreed with its use upon explanation (quote, Table 2). A total of three participants with the diagnosis of NRFP chose to use a term other than “NRFP.” NRFP patients frequently used “migraine” and “sinus migraine” to describe their symptoms, suggesting they distinguished between CRS and their specific set of symptoms. All participants diagnosed with CRS understood the term and preferred its use in the interviews.

#### How patients describe facial pain, pressure, and discomfort

Facial pain, pressure, and discomfort are hallmark symptoms of both CRS and NRFP. Therefore, interviews began by asking participants to describe their experiences with pain, pressure, or discomfort on their face, head, and neck. All participants in both diagnosis groups were able to describe their pain clearly and confidently. Many participants in both groups used the terms “pressure” and “pain” to describe their discomfort. The location of pain and pressure was similar among both diagnosis groups: around their noses, eyes, forehead, jaw, and neck.

#### Description of symptom experiences

Participants were asked, “How would you describe a typical episode [of NRFP/sinusitis]?” The most common symptoms of typical episodes among NRFP patients were facial pressure and pain, fullness, congestion, and dizziness. Common symptoms during a typical episode among participants with CRS were sinus pressure, headache, and stuffy nose (quotes, Table 2).

**Fig. 3:**
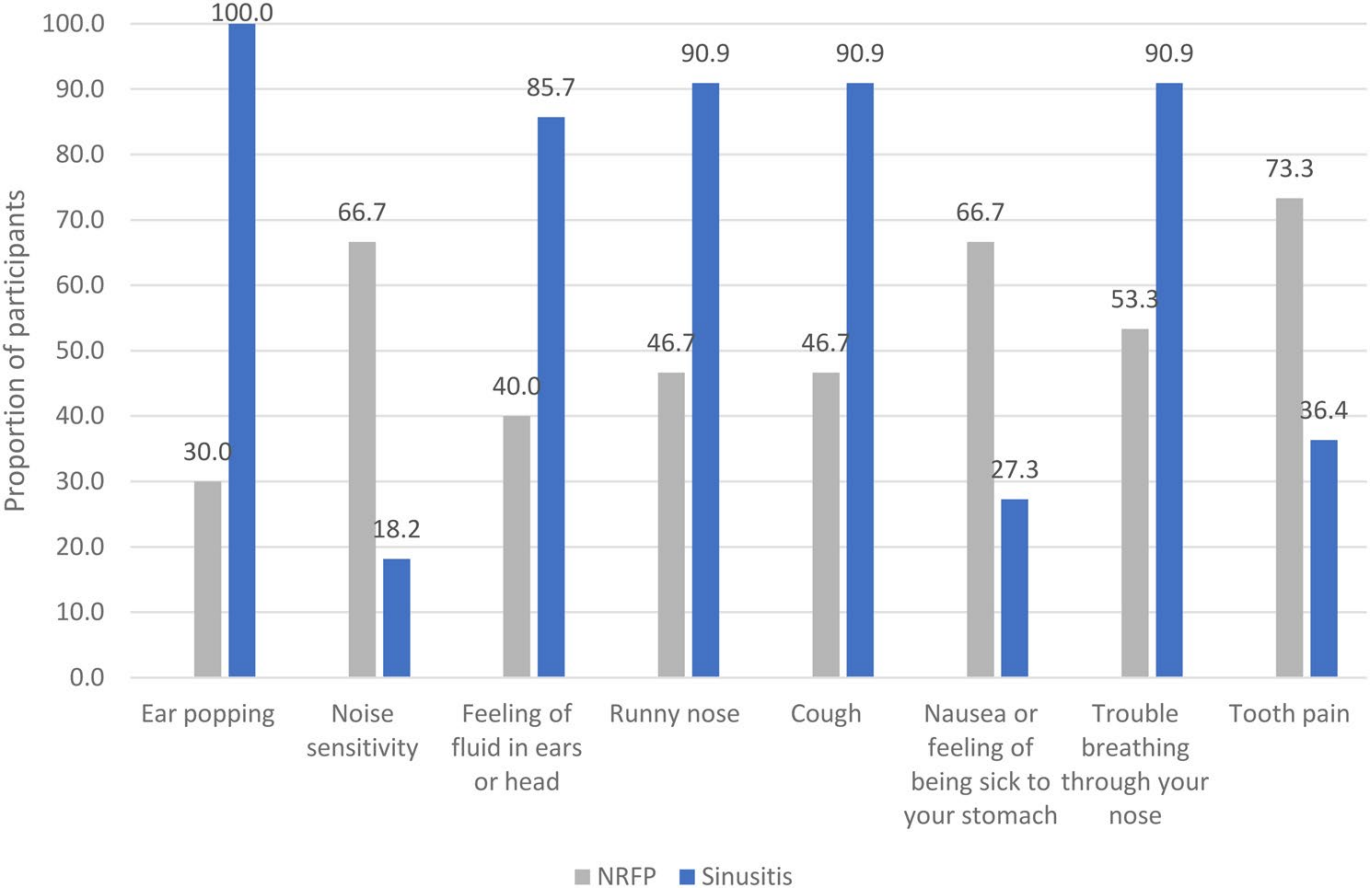
Symptoms by diagnosis for symptoms with greater than 35% difference in reporting from the qualitative sample

All symptoms reported by participants were tabulated; the number of participants and proportion of participants reporting symptoms by diagnosis are presented in Appendix 6. Figure 3 shows symptoms that were most differently experienced by diagnosis. For example, ear popping was mentioned by all participants diagnosed with CRS (100%) but by only 30% of participants diagnosed with NRFP. More participants with NRFP reported experiencing noise sensitivity, nausea, and tooth pain compared to participants with rhinosinusitis.

#### Episodic or chronic nature of symptoms

Participants reported that they generally experienced the same constellation of symptoms with each episode. Those classified as having NRFP were more likely to report shorter episode lengths (less than 1 day), while those with CRS reported that most episodes lasted a few days to a month or chronically. No NRFP participants indicated chronic symptoms in concept elicitation interviews. Seven participants with CRS expressed having their symptoms present most of the time. During these chronic cases, participants experienced either consistent pain and pressure or daily episodes (quote, Table 2).

#### Differentiating types of episodes

Given the episodic nature of some patient experiences, patients may or may not be experiencing symptoms when they visit the clinic. Therefore, we investigated how best to capture symptom experiences for the purposes of screening out CRS. We inquired about participants’ most recent episode, typical episode, and most-severe episode. All but one participant described their most recent episode as a typical episode.

Participants were asked what characterizes severe symptom episodes. Participants were easily able to differentiate a typical episode from a severe episode. The most notable difference between typical and most severe episodes for both groups was in pain intensity. Ten of thirteen NRFP participants indicated that their pain intensified during their most severe episodes. While increased pain intensity was mentioned by most of those with NRFP, nausea was also mentioned in five of thirteen responses. Seven of ten participants with CRS mentioned increased pain during their most severe episodes. Six of ten also reported increased congestion and other sinus symptoms such as postnasal drip, stuffy nose, and runny nose (quote, Table 2).

#### Symptoms outside of episodes

After round 1, a clinical panel member indicated that symptoms outside of episodes might be an indicator of NRFP. In rounds 2 and 3, participants were asked what symptoms they experienced outside of episodes. The most common symptoms experienced outside of episodes by participants with NRFP were congestion, brain fog, and fatigue. For participants with CRS, the most common symptoms outside of episodes were congestion, headache, watery or teary eyes, blurry or hazy vision, and dizziness.

#### Treatments that work

In rounds 2 and 3, participants were asked which medications or treatments help their symptoms in order to examine possibly adding items about treatment efficacy. Not all participants tried each type of medication. Most participants with NRFP who tried migraine medications indicated that migraine medications improved their symptoms (6/7). Over-the-counter pain relievers were indicated as helpful in addressing symptoms for most of individuals with NRFP (6/10). For participants with CRS, the most frequently reported treatment identified as useful in addressing symptoms was oral steroids (5/7). Antibiotics were reported as helpful in addressing symptoms by 4/7 of CRS participants and 3/7 NRFP participants. Other useful treatments for CRS participants were antihistamines (4/7) and over-the-counter pain relievers (4/7).

#### Triggers of symptoms/episodes

Participants were asked about triggers for their episodes. For those with NRFP, the most frequently reported triggers were stress, poor sleep, allergies, and changes in environmental barometric pressure (quote, Table 2). For those with CRS, the most common triggers were stress, allergies, and changes in environmental barometric pressure. In addition to these, for CRS participants in particular, having a cold was an important trigger for their CRS symptoms.

#### Family and childhood history

Family and childhood history with migraine-like symptoms was elicited in round 1 to develop items for testing in cognitive interviews. A slightly higher proportion of individuals with NRFP reported a history of migraines in the family or history of headaches as a child (Appendix 7).

**Table 2:** Concept elicitation illustrative quotes from participants

Concept characteristic	Participant quote	Participant characteristic
Terms used to describe diagnoses: *sinus migraine*	“So, it distinguishes from just like a regular migraine. When I say it, people understand that it’s related to my sinus issues, and it makes sense.”	#109, NRFP, female, Black or African American, not Hispanic/Latino, age ≥ 60
Description of symptom experiences: *typical symptoms*	“Typically, it is a combination of the sinus pain in conjunction with my ears ringing. Sinus pain alone isn’t enough for me to take the medicine. Last night I was having sinus pain – I’m sorry, it’s really pressure. The pain only comes if I’m really dry as well – but sinus pressure, and I had the ear ringing really loudly last night. And I took one of the sublingual medicine for the headaches, and I’m not quite sure it went away.”	#107, NRFP, female, White, not Hispanic/Latino, age 18–59
	“The pressure, the congestion that won’t move. It’s like unproductive congestion. And it just drains in the back of my throat, which will eventually, if I don’t see about it, cause a bad cough… Usually it’s just – for my normal – it’s gross, just normal daily mucus or pressure. It’s not – my teeth aren’t hurting. Oh, oh. I forgot to say swelling too because I know I have a fat, chunky face, but I can tell when it’s swollen in certain areas. So, when I’m having an episode, it’s swollen; it’s more tender to the touch. When I’m not having an episode, it’s pressure and pain but not as bad as it is when my sinuses are flaring up.”	#108, NRFP, female, Black or African American, not Hispanic/Latino, age 18–59
	“It typically starts by something that would trigger it. It could be – I don’t know – sometimes I feel that maybe it is season-related, if I go outside during the particular times. Or I want to walk fast to reach a destination it starts by just – okay. So, when they start it is sort of like a sharp, shooting pain in my forehead and behind the eyes. And I cannot breathe through my nose at all. It’s basically nothing goes in or comes out. Then I have to breathe through my mouth. That itself will, of course, increase my heartrate. And then the pain and the pressure start to become very significant. Before my treatments, at this stage I would start to sneeze and cough a lot as well. And now that I had my treatments the sneezing and the coughing is not as much. So, I would say maybe I would sneeze once or twice. But the coughing could still be there associated with this type of episode.”	#210, CRS, female, White, not Hispanic/Latino, age18-59
Episodic or chronic nature of symptoms	“Well, I think I just described it just the pain was chronic, the headache was chronic, and just the stuff in my nose. And there was just a lot of postnasal drip, thick, and multicolored, and nasty, and it wasn’t really a come and go thing. It was just always there and that was part of the problem is that there was just no relief from it.”	#204, CRS, female, White, not Hispanic/Latino, age ≥ 60
Differentiating types of episodes: *worst episode*	“So, when it’s at its worst – like the absolute worst – … I will oftentimes describe it is I’ll wake up in the morning, I’ll flip on the light in the bathroom and be like, “Nope.” Flip the light back on and crawl right back into bed.”	#114, NRFP, female, White, not Hispanic/Latino, age 18–59
Triggers of symptoms/episodes	“I have a lot of allergies and – when my allergies kick in – it’s all sinus every single time. I don’t care if it’s the pollen or if it’s perfume or powders. Spices, there are some spices – a combination of – that will trigger it. Other than that, I mean I can’t tell. But once it triggers, it’s there.”	#101, NRFP, female, White, not Hispanic/Latino, age ≥ 60

Abbreviations: CRS = chronic rhinosinusitis; NRFP = non-rhinogenic facial pain

### Part 2: Cognitive interview results

Results of the cognitive interviews are described here, with illustrative quotes from participants in Table 3.

**Fig. 4:**
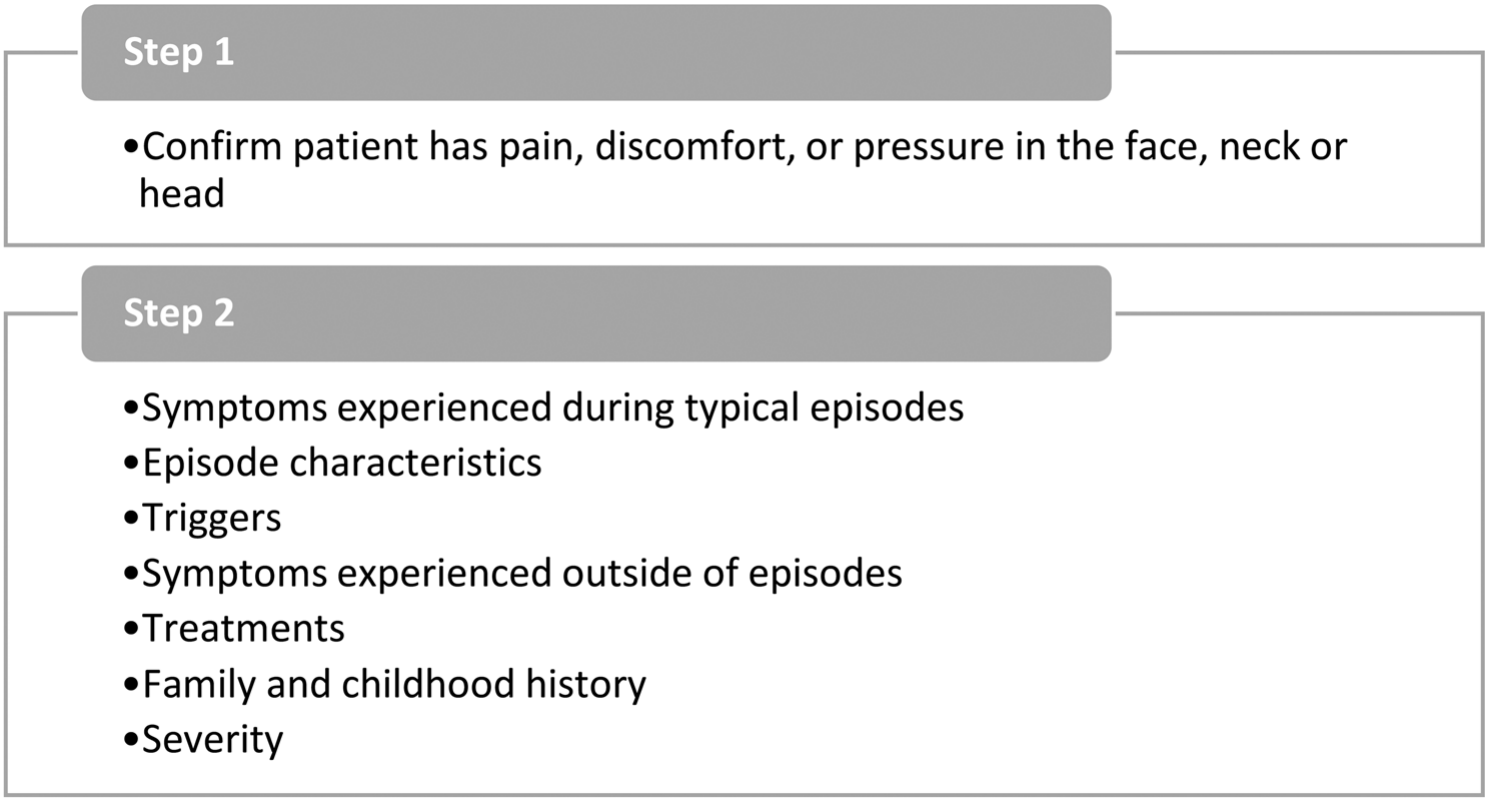
Screening questionnaire sections

The cognitive interviews explored sections of the screening questionnaire separately (Fig. 4). The screening questionnaire was deliberately designed as a 2-step screener to manage patient burden. The first question identifies patients with pain, discomfort, or pressure in their face. Only patients who experienced these symptoms proceed to the rest of the screening questions.

The next sections describe key changes made to items during the cognitive interviews. Round 3 was paused after 2 interviews due to compelling findings that required changes. A full item tracking matrix is presented in Appendix 4, including a step-by-step description of what changed and why between each round (and during round 3).

#### Step 1

The first question in the screening questionnaire confirms that the participant has facial pain. This question went through two primary changes throughout the cognitive interview rounds to address issues identified during the interviews. First, the stem of the question changed from “Are you seeing your doctor today for…” to “What are the reasons you are seeing your doctor today?” The reason for this change is that patients could present with other symptoms in addition to facial pain/discomfort, and the item was modified to give them an opportunity to indicate the reasons they are visiting their doctor. Only patients who experience facial pain/pressure continued to step 2 of the screener. The second change was to include additional parts of the head based on participants’ descriptions of the location of their discomfort, including eyebrows, jaw, and teeth.

#### Step 2

##### Symptoms

Most participants in both NRFP and CRS groups reported that it was easy to answer the symptom questions. The choice of recall period for the symptom questions was rooted in concept elicitation findings that participants could easily describe their typical episode. The format of the symptom question changed from a yes/no question to a 5-point rating scale (“Always,” “Very often,” “Sometimes,” “Rarely,” “Never,” “I don’t know”). The reason for this change was that multiple participants indicated it was difficult to choose between binary responses (yes vs. no) or even a 3-point scale. The instructions changed between rounds to address confusion from participants who had chronic symptoms, and whether to include side effects of medications when responding to the symptom question. Participants did not have trouble differentiating medication side effects from symptoms in their typical episodes (quote, Table 3).

Between rounds, symptoms were added such as ear popping, twitching on face, brain fog, dry eyes (see Item Tracking Matrix in Appendix 4 for details). Most participants indicated that the table format was intuitive, and most chose symptoms in the table that they shared during concept elicitation. Some participants identified symptoms that they experienced but forgot to mention in concept elicitation (quotes, Table 3).

##### Episode characteristics

Round 1 focused on open-ended concept elicitation questions to characterize the length of episodes. Between round 1 and round 2, an item was developed to capture episode length from less than 24 h to more than 2 weeks based on responses from round 1. During round 2, participants with chronic symptoms had trouble choosing a response, thus “Chronic symptoms - daily or almost daily” was added as a response choice. Round 3 confirmed that the item was working as intended and no additional changes were made. Another item in the Episode Characteristics section of the screener sought to characterize participants’ mucus during typical episodes. Between round 1 and round 2, a change was made to add a response choice for patients who did not have mucus attributed to their condition during their typical episodes. No other changes were made to this item in subsequent rounds.

##### Triggers

The section on triggers was derived between round 1 and round 2 based on triggers elicited during round 1. During round 2, additional triggers were identified that were not already included in this question and were added as response choices. Examples of triggers added include humidity, menstrual cycle, and alcohol.

##### Symptoms experienced outside of episodes

During round 1, participants brought up symptoms outside of episodes during concept elicitation. Study clinicians hypothesized that consistent experiences outside of episodes may help differentiate CRS from NRFP, thus this section was added to capture symptoms outside of episodes based on elicited information in round 1. Symptoms included congestion, headache, and sensitivity to sounds or light. After round 2, only one symptom was added, ear fullness. Response choices were modified from yes/no to a 4-point scale to capture the full range of participants’ experiences: “Always or most of the time outside of episodes,” “Sometimes outside of episodes,” and “I do not experience this symptom outside of episodes,” and “I experience this symptom chronically.”

##### Treatments

The section on treatments was developed to examine treatment effectiveness as a potentially important piece of information to differentiate CRS from NRFP. The treatment questions began as individual questions for each treatment type. Between round 1 and round 2, items were modified due to various issues, such as a double-barreled question, and participants not being familiar with medication names. Based on participant feedback, the treatment questions were transformed to a tabular format, and treatments were added to the table based on participants mentioning treatments that were not already included in the table. Examples of treatments added were oral steroids, caffeine, and over-the-counter pain relievers. By round 3, the treatment item was stable and no additional issues were identified.

##### Family and childhood history

Family and childhood history of migraine-related symptoms were explored during concept elicitation in round 1. Rounds 2 and 3 tested three items: family history of unexplained headaches, childhood headaches, and motion sickness as a child. No issues were identified in the cognitive interviews for these items.

##### Severity

Response choices were changed for the question asking about the level of discomfort on patients’ face, neck, or head. Response choices were changed from a verbal rating scale to a numeric rating scale due to patients having trouble choosing a response with each response having a verbal description. After round 1, an item was added to assess what extent typical episodes limited participants’ activities. After this item was added, no issues were identified in the remaining rounds.

##### Addressing chronic symptoms

Participants with chronic symptoms had trouble answering questions about their typical episodes. Therefore, modifications were made to the screening questionnaire to ensure experiences were captured without asking participants to answer questions that did not apply to them (e.g., symptoms between episodes). Approaches to address chronic symptoms included skip logic, and additional response choices indicating that some experiences are chronic.

##### Open-ended responses

To ensure that the screening questionnaire comprehensively captured relevant experiences of patients with CRS and NRFP, we included open-ended responses after questions to capture experiences that were not identified in the qualitative study. After the screening questionnaire has been administered in multiple prospective studies and we are confident in the comprehensiveness of the response choices, we plan to eventually remove the open-ended responses.

**Table 3:** Cognitive interview illustrative quotes from participants

Question	Participant quote	Participant characteristic
Symptoms: *differentiating side effects from symptoms*	“Well, I have to go through all the side effects before I can tell that [migraine medication] helps. So, that’s what goes on within that 45 min to an hour. So, it does take about an hour. You feel so crummy within that time because of the pill that at the end of the hour you say, “Oh it did get rid of the headache.”	#111, NRFP, female, White, not Hispanic/Latino, age ≥ 60
Symptoms: *tabular format*	“Well, it’s visually more simple than just sentences written out without borders. So, it’s actually pretty straightforward and it’s easy to visually understand each line is a different question”.	#103, NRFP, female, White, not Hispanic/Latino, age 18–59
Symptoms: *comprehensiveness of table*	“Well, it’s funny, there was a couple things here I forgot all about, like the taste, smell.”	#207, CRS, male, White, not Hispanic/Latino, age ≥ 60

Abbreviations: CRS = chronic rhinosinusitis; NRFP = non-rhinogenic facial pain

All questions developed for the SHS tool are presented in Appendix 8.

## Discussion

This study aimed to develop a list of potential items for the SHS based on qualitative interviews with patients diagnosed with NRFP or CRS. The list of SHS items consists of 8 sections and 89 items (Fig. 4). The qualitative data provided robust evidence for assessing typical episodes, a distinctive component of this screening tool. Participants could clearly articulate their typical episodes, distinguishing them from the most severe or other types of episodes. They were also able to describe the consistency of symptoms during episodes (symptom constellations) and the episodic or chronic nature of their symptoms. This insight enabled the creation of questions focused on typical episode characteristics.

For participants with constant symptoms, the questionnaire incorporates skip logic to bypass irrelevant questions about triggers and episodic symptoms. The symptom list was extensive, with 46 symptoms identified. Notably, even within this qualitative sample, symptom experiences varied between CRS and NRFP patients, with noise sensitivity, nausea, and tooth pain being more prevalent in those with NRFP. Given that NRFP is not yet a formal diagnosis, it was expected that participants were unfamiliar with the term. Preliminary evidence suggests that family and childhood history are unlikely to be significant indicators of NRFP.

The majority of participants were female, reflecting the higher prevalence of migraine symptoms among women. Strengths of this study include the systematic approach to developing the screening tool, incorporating expert panel input, concept elicitation, and cognitive interview rounds. The methodological approach of combining concept elicitation and cognitive interview questions into each interview accelerated item development while still providing an opportunity to document new concepts elicited from participants. This approach was appropriate for the development of PROM for screening because the goal is to ensure *key* NRFP and CRS concepts are included in the measure, but winnowing of items is left to the quantitative phase when criterion validity assessments occur. Beyond CRS and NRFP, this study provides evidence of the feasibility of this approach in the development of other PROMs for screening. The (forthcoming) quantitative validation study will examine evidence of sensitivity and specificity of the SHS for differentiating NRFP from CRS after item pruning. Additionally, the a priori–defined diagnostic criteria for NRFP ensured representation of individuals with both diagnoses in the sample, and purposive sampling promoted diversity in participant characteristics.

However, the study has limitations. First, participants were recruited from a single health system, interviews were conducted only in English, and despite purposive sampling, the team could not enroll participants of Spanish/Hispanic/Latino ethnicity within the timeline and budget allotted for this study. Future research should expand across multiple institutions and include a Spanish version of the questionnaire for a broader representation of the U.S. population. Second, the sample was largely female. Though NRFP is more prevalent in women, illustrative quotes identified in the concept elicitation portion of the interviews were all from women. Third, all but one interviews were conducted between 9 AM and 5 PM. Participants were asked for their availability and advised that interviews could be scheduled outside of typical business hours; however, it is possible the study missed perspectives from people who were unavailable during the day.

This study represents the first important step in developing a screening questionnaire, the SHS, to flag patients with NRFP in clinical settings. Such a questionnaire stands to prevent the use of unnecessary antibiotics and guide patients to the care they need sooner to reduce suffering. Future research will involve prospective data collection in rhinology clinics to identify the most effective questions for differentiating between CRS and NRFP, aiming to refine the questionnaire to maximize sensitivity for NRFP identification. The subsequent step is to assess criterion validity of the questionnaire in primary care settings.

## Supplementary Information

Below is the link to the electronic supplementary material.

Supplementary Material 1

Supplementary Material 2

Supplementary Material 3

Supplementary Material 4

Supplementary Material 5

Supplementary Material 6

Supplementary Material 7

Supplementary Material 8

## Data Availability

The data that support the findings of this study are available from the corresponding author upon reasonable request.

## References

[1] Eross E, Dodick D, Eross M (2007) The Sinus, allergy and migraine study (SAMS). Headache 47(2):213–224. 10.1111/j.1526-4610.2006.00688.x 17300361 10.1111/j.1526-4610.2006.00688.x

[2] Schreiber CP, Hutchinson S, Webster CJ, Ames M, Richardson MS, Powers C (2004) Prevalence of migraine in patients with a history of self-reported or physician-diagnosed sinus headache. Arch Intern Med 164(16):1769–1772. 10.1001/archinte.164.16.1769 15364670 10.1001/archinte.164.16.1769

[3] Dekker AR, Verheij TJ, van der Velden AW (2015) Inappropriate antibiotic prescription for respiratory tract indications: most prominent in adult patients. Fam Pract 32(4):401–407. 10.1093/fampra/cmv019 25911505 10.1093/fampra/cmv019

[4] Jang DW, Lee HJ, Chen PG, Cohen SM, Scales CD (2021) Geographic variations in healthcare utilization and expenditure for chronic rhinosinusitis: A Population-Based approach. Laryngoscope 131(12):2641–2648. 10.1002/lary.29588 33904602 10.1002/lary.29588

[5] Bhattacharyya N, Lee LN (2010) Evaluating the diagnosis of chronic rhinosinusitis based on clinical guidelines and endoscopy. Otolaryngol Head Neck Surg 143(1):147–151. 10.1016/j.otohns.2010.04.012 20620634 10.1016/j.otohns.2010.04.012

[6] Rapoport AM, Bigal ME (2004) ID-migraine. Neurol Sci 25(Suppl 3):258–260. 10.1007/s10072-004-0301-9 10.1007/s10072-004-0301-9 15549552

[7] Agius AM (2010) Long-term follow-up of patients with facial pain in chronic rhinosinusitis–correlation with nasal endoscopy and CT. Rhinology 48(1):65–70. 10.4193/Rhin09.015 20502737 10.4193/Rhin09.015

[8] Jones NS (2004) Midfacial segment pain: implications for rhinitis and sinusitis. Curr Allergy Asthma Rep 4(3):187–192. 10.1007/s11882-004-0025-1 15056400 10.1007/s11882-004-0025-1

[9] Schulz KA, Esmati E, Godley FA, Hill CL, Monfared A, Teixido M, Tucci DL, Witsell DL (2018) Patterns of migraine disease in otolaryngology: A CHEER network study. Otolaryngol Head Neck Surg 159(1):42–50. 10.1177/0194599818764387 29558248 10.1177/0194599818764387

[10] Patrick DL, Burke LB, Gwaltney CJ, Leidy NK, Martin ML, Molsen E, Ring L (2011) Content validity–establishing and reporting the evidence in newly developed patient-reported outcomes (PRO) instruments for medical product evaluation: ISPOR PRO good research practices task force report: part 1–eliciting concepts for a new PRO instrument. Value Health 14(8):967–977. 10.1016/j.jval.2011.06.014 22152165 10.1016/j.jval.2011.06.014

[11] Willis G (2005) Cognitive interviewing: a tool for improving questionnaire design. Sage

[12] Abdul Rehman A, Alharthi K (2016) An introduction to research paradigms

[13] Guest G, Bunce A, Johnson L (2006) How many interviews are enough? An experiment with data saturation and variability. Field Methods 18(1):59–82

[14] Patton M (2015) Qualitative research and evaluation methods. 4th Ed. Sage Publications

[15] Rosenfeld RM, Piccirillo JF, Chandrasekhar SS, Brook I, Ashok Kumar K, Kramper M, Orlandi RR, Palmer JN, Patel ZM, Peters A, Walsh SA, Corrigan MD (2015) Clinical practice guideline (update): adult sinusitis. Otolaryngol Head Neck Surg 152(2 Suppl):S1–s39. 10.1177/0194599815572097 25832968 10.1177/0194599815572097

[16] Hsieh HF, Shannon SE (2005) Three approaches to qualitative content analysis. Qual Health Res 15(9):1277–1288. 10.1177/1049732305276687 16204405 10.1177/1049732305276687

