# Supplementary material for: Development of the Sinus Headache Screener to identify patients with non-rhinogenic facial pain compared with chronic rhinosinusitis in rhinology clinics
Source: J Patient Rep Outcomes. 2025 Nov 6;9:130. doi: 10.1186/s41687-025-00956-4 (PMC12592570; doi:10.1186/s41687-025-00956-4)
Supplement: Supplementary file 1 — Supplementary Material 1 [file 41687_2025_956_MOESM1_ESM.docx]

**Appendix 1. Prestudy Screening Questionnaire**

*Purpose: Study screening questionnaire to determine eligibility and obtain a balanced sample*

Please answer the following questions, as they best describe your situation.

1. What is your age? ___ [If less than 18 years – ineligible]

| 2. What is your gender? □_1_ Man □_2_ Woman  □_3_ Self Identify: ______________ □_4_ Prefer not to say |
| --- |
|  |
| 3. Are you of Spanish/Hispanic/Latino origin? □_1_ No □_2_ Yes |
|  |
| 4. What is your racial or ethnic background? *(Please check all that apply)*  □_1_  White  □_2_ Black or African-American  □_3_  American Indian or Alaska Native  □_4_ Asian  □_5_ Native Hawaiian or other Pacific Islander  □_6_ Prefer not to answer |

5. What is the highest grade in school that you completed?

- _1_ Less than high school
- _2_ High school graduate or equivalent (e.g., GED)
- _3_ Completed some college, but no degree
- _4_ Completed associate degree/diploma program
- _5_ College graduate (e.g., B.A., B.S.)
- _6_ Completed graduate school

6. How would you like to participate in the interview?

- Phone
- Zoom or webex

6a. [*If Zoom or webex was chosen:*] Do you have access to a laptop or tablet that you could use to participate in interviews?

- Yes
- No

[*If no:* OK, we can complete the interview via phone. We’ll need to send you some documents via hard copy.]

7. How would you like to be contacted for reminders about the interview? (Indicate all that apply.)

- Phone
- Email
