# Supplementary material for: Development of the Sinus Headache Screener to identify patients with non-rhinogenic facial pain compared with chronic rhinosinusitis in rhinology clinics
Source: J Patient Rep Outcomes. 2025 Nov 6;9:130. doi: 10.1186/s41687-025-00956-4 (PMC12592570; doi:10.1186/s41687-025-00956-4)
Supplement: Supplementary file 2 — Supplementary Material 2 [file 41687_2025_956_MOESM2_ESM.docx]

**Appendix 3. Concept Elicitation Codebook**

| Structural Codes | Mid-level Structural Codes |
| --- | --- |
| Term for Condition - [IDI question: Does the term, XXX seem fitting for what I described?] |  |
| Description of Symptoms - [Includes description of Pain, Pressure, Discomfort and other symptoms they experience. IDI question: Please describe your experience with discomfort to me in your own words.] |  |
| Term for Pain, Presure or Discomfort (shooting, throbbing, etc.) |  |
| Frequency and Consistency of Symptoms - [IDI question: Do you symptoms come and go, or are they around most of the time?] |  |
| Term for Symptom Experience (episodes, etc.) |  |
| Description of Typical Episodes |  |
|  | **Severe or Typical Episode** - includes Difference Between Typical and Severe what makes an episode typical compared to severe episode |
|  | **Typical or Most Recent Episode-** includes Difference Between typical and most recent **episode** what makes an episode typical compared to most recent episode |
| Description of Beginning and End of Episodes- [IDI question: how do you know when a typical episode is starting and ending?] |  |
| Improvement in Experience - [IDI question - If there was a new medication available to improve your experiences with XXX what aspects of XXX are important to be changed] |  |
