# Supplementary material for: Development of the Sinus Headache Screener to identify patients with non-rhinogenic facial pain compared with chronic rhinosinusitis in rhinology clinics
Source: J Patient Rep Outcomes. 2025 Nov 6;9:130. doi: 10.1186/s41687-025-00956-4 (PMC12592570; doi:10.1186/s41687-025-00956-4)
Supplement: Supplementary file 3 — Supplementary Material 3 [file 41687_2025_956_MOESM3_ESM.docx]

| **QUESTION** | **R1 Version** | **R1 Description of problem** | **R2 Version** | **R2 Description of the problem** | **R3** | **R3v1 description of the problem** | **R3v2** | **R3v2 description of the problem** | **Final modifications** |
| --- | --- | --- | --- | --- | --- | --- | --- | --- | --- |
| **1** | Are you seeing your doctor today for discomfort anywhere on your face, neck or head (including eyes, ears, cheeks, temples, or nose)? | A variety of terms are being used to describe patients' discomfort, but all participants understood the question as intended. Eyebrows and jaw were added as areas of the head that could be included based on participants' descriptions of the location of their discomfort. | Are you seeing your doctor today for discomfort anywhere on your face, neck, or head (including eyes, ears, cheeks, **eyebrows, jaw** , temples, or nose)? | Some participants did not use the term discomfort to describe their episodes. Teeth pain can be observed in both conditions | Are you seeing your doctor today for discomfort, **pain, or pressure** anywhere on your face, neck, or head (including eyes, ears, cheeks, eyebrows, jaw, temples, **teeth**, or nose | Concern from the study team that the main reason for seeing the dr may not be the discomfort, pain or pressure experienced by the patient; it could be another symptom. Therefore, the item was modified to allow for multiple conditions. | ~~Are you seeing your doctor today for discomfort, pain, or pressure anywhere on your face, neck, or head (including eyes, ears, cheeks, eyebrows, jaw, temples, teeth, or nose~~  What are the reasons you are seeing your doctor today? (Please check all that apply.) |  |  |
| **2** | During a typical episode, do you have… (check one response per row) | A number of participants indicated that it was difficult to choose between yes, no and that more response choices would be helpful | Response choices modified from yes, no, I don't know to **Always or most of the time; sometimes; never; I don't know** |  |  | Expanded response choices to account for patients having trouble choosing among the 3-point scale, and clinical team feedback | Response choices modified to Always ~~or most of the time~~; **very often**; sometimes; **rarely**, never; I don't know | Feedback from stakeholder panel was to group like-symptoms together | items were grouped by ear, face, mouth, nausea, and sensation symptoms |
|  |  | Participants had trouble answering this question when their symptoms were chronic. | Sentence added to instructions: If your episodes are chronic, please think about those symptoms when answering these questions. | Participants sometimes included symptoms they experienced as side effects of treatment | Sentence added to instructions: Please do not answer about side effects you may experience due to medications for your condition. |  |  |  |  |
|  | nausea or feeling of being sick to your stomach |  |  |  |  |  |  |  |  |
|  | vomiting |  |  |  |  |  |  |  |  |
|  | trouble breathing through your nose |  |  |  |  |  |  |  |  |
|  | dizziness or trouble with balance |  |  |  |  |  |  | patients indicated they would respond differently about dizziness vs trouble with balance; separated to two items | dizziness |
|  | lightheadedness |  |  |  |  |  |  | lightheadedness was brought up by 2 patients as a unique symptom | lightheadedness |
|  | post-nasal drip |  |  |  |  |  |  |  |  |
|  | new or worsening runny nose | The term "new or worsening" was confusing to participants in relationship to a "typical episode". | ~~new or worsening~~runny nose |  |  |  |  |  |  |
|  | new or worsening stuffy nose | The term "new or worsening" was confusing to participants in relationship to a "typical episode". | ~~new or worsening~~ stuffy nose |  |  |  |  |  |  |
|  | bright flashes of light that no one else saw |  |  |  |  |  |  |  |  |
|  | an experience of smelling odors that no one else smells |  |  |  |  |  |  |  |  |
|  | numbness in your face |  |  |  |  |  |  |  |  |
|  | tingling in your face |  |  |  |  |  |  |  |  |
|  | ringing in one or both ears |  |  |  |  |  |  |  |  |
|  | ear pain |  |  |  |  |  |  |  |  |
|  | ear fullness |  |  |  |  |  |  |  |  |
|  | headache |  |  |  |  |  |  |  |  |
|  | teary or watery eyes |  |  |  |  |  |  |  |  |
|  | an experience of light touch being painful |  |  |  |  |  |  |  |  |
|  | pain when chewing |  |  |  |  |  |  |  |  |
|  | light sensitivity |  |  |  |  |  |  |  |  |
|  | noise sensitivity |  |  |  |  |  |  |  |  |
|  | sore throat |  |  |  |  |  |  |  |  |
|  | green, yellow, or brown nasal discharge from nose |  |  |  |  |  |  |  |  |
|  | cough |  |  |  |  |  |  |  |  |
|  | tooth pain |  |  |  |  |  |  | some patients thought of the tooth sensation as pain, others thought of it as sensitivity | tooth pain or sensitivity |
|  | sensitivity to smells |  |  |  |  |  |  |  |  |
|  | sensitivity to taste |  |  |  |  |  |  |  |  |
|  | foul taste in mouth |  |  |  |  |  |  |  |  |
|  |  | added based on interviews | felt nauseated when reading in a moving vehicle |  |  |  |  | some patients did not read in cars during episodes, or had motion sickness without reading; item was separated into two items |  |
|  |  |  |  |  |  |  |  | added due to reason above | motion sickness |
| **3** | What was your level of discomfort anywhere on your face, neck, head (including eyes, ears, cheeks, temples, or nose) during your most typical episodes? | participants had trouble answering using the answer choices: no discomfort, mild, moderate, severe, very severe. Participants were able to describe their pain on a 5-point scale. Also participants typically used the term "pain' to describe their discomfort. | How would you rate your level of discomfort on your face, neck, or head (including eyes, ears, cheeks, eyebrows, jaw , temples, or nose)? |  |  |  |  |  |  |
|  |  | study team wanted to know how much participants' discomfort limited their activities | To what extent do your typical episodes limit your activities? |  |  |  |  |  |  |
| **4** | During your typical episodes, how would you describe your mucus/snot? (Please check all that apply.) | there was no response option for people who did not have mucus during their episodes | changed one of the response options: I do not have **mucus**, runny nose, or congestion during my episodes. |  |  |  |  | Added a response choice for individuals who are so congested that they are unable to describe the consistency of their mucus | **I am unable to determine the consistency of my mucus/snot during my episodes** |
| **5** | How often are you treated with antibiotics for your episodes? | Potentially not a helpful question because we are mostly interested in whether the antibiotics worked. | Removed item |  |  |  |  |  |  |
|  |  | research team thought treatments should be in a table format and remove the yes/no response option | Do any of the following help to address symptoms during your typical episodes? (check one response per row) | Patients indicated that the medications listed may help some symptoms of their episodes, but not all symptoms in their episode. | Do any of the following help to address **some or all of your** symptoms during your typical episodes? (check one response per row) |  |  | Per stakeholder feedback, add another treatment, "Nasal saline rinse" | Nasal saline rinse |
|  |  |  | Response choices were modified to **Always or most of the time, Sometimes, No, I have not tried these** |  |  | Response choices were expanded because participants had a hard time with the 3-point scale; felt it would be easier to respond with more detail | Response choices were modified to Always ~~or most of the time~~, Very often Sometimes, **Rarely**, **Never** ~~No~~, I have not tried these |  |  |
| **6** | Do your episodes get better when you take medicine for allergies (such as Flonase or an antihistamine)? | Double barrelled question - one participant indicated that they would answer differently depending on the medication class they were thinking about. This item was broken into two items | nasal spray (such as Flonase)? | clinical team thought it was best to specify the type of nasal sprays since they can be decongestants and antihistamines | Response choice modified to nasal **steroid** spray (such as Flonase, **Nasonex**)? |  |  |  |  |
|  |  |  | Antihistamines (such as Benadryl, Claritin, Zyrtec, Allegra) |  |  |  |  |  |  |
| **7** | Do your episodes get better when you take “Triptan”, Sumatriptan, or Imitrex? | Some participants had trouble with this question if they were not familiar with these medications. Therefore, a clause was added to indicate that these are migraine medications | Do your episodes get better when you take **migraine medications such as** “Triptan”, Sumatriptan, or Imitrex? |  |  |  |  |  |  |
| **8** | Do your episodes get better when you take antibiotics |  | antibiotics |  |  |  |  |  |  |
|  |  | Added due to participants mentioning them in interviews | Oral steroids |  |  |  |  |  |  |
|  |  | Added due to participants mentioning them in interviews | Caffeine |  |  |  |  |  |  |
|  |  | Added due to participants mentioning them in interviews | Ove-the-counter pain reliever (such as Advil, Tylenol) |  |  |  |  |  |  |
|  |  |  |  | paticipants mentioned decongestants in R2 interviews | Decongestants (such as SudafedClaritin-D, Allegra-D, Afrin ) |  |  |  |  |
|  |  |  |  | one participant mentioned injectable medication | Injectable medicati ons (such as Dupixant) |  |  |  |  |
|  |  | Added new question based on clinical team insights about migraine patients | Do you have any family history of migraine or unexplained headaches? |  |  |  |  |  |  |
|  |  | Added new question based on clinical team insights about migraine patients | Did you recall having unexplained headaches as a child? |  |  |  |  |  |  |
|  |  | Added new question based on clinical team insights about migraine patients | Did you recall having motion sickness as a child? |  |  |  |  |  |  |
|  |  | Added a question based on responses during the concept elicitation section of the interviews | How long do your typical episodes typically last? | Participants with chronic episodes had a hard time picking a response for length of episodes | added the following response option: Chronic symptoms (daily or almost daily) |  |  | A number of patients experienced episodes for less than 7 days; added response choices to be more specific | Less than 24 hours; **1-3 days; 4-7 days**; 8-14 days; More than 2 weeks; chronic symptoms (daily or almost daily) |
|  |  | Added based on participants' responses to questions on triggers | Do any of the following trigger an episode? |  |  |  |  |  |  |
|  |  |  | Stress |  |  |  |  |  |  |
|  |  |  | Poor sleep |  |  |  |  |  |  |
|  |  |  | smells |  |  |  |  |  |  |
|  |  |  | Allergies (seasonal and pets) |  |  |  |  |  |  |
|  |  |  | Changes in pressure | added based on the way this trigger was expressed in interviews | ~~Changes in pressure~~ Daily weather changes |  |  | weather changes was confusing to patients and they more-often talked about change in pressure due to weather; re-worded item | **changes in pressure due to weather** |
|  |  |  | Having a cold |  |  |  |  |  |  |
|  |  |  |  | added based on triggers mentioned in interviews | Humidity |  |  |  |  |
|  |  |  |  | added based on triggers mentioned in interviews | Menstrual cycle |  |  |  |  |
|  |  |  |  | added based on triggers mentioned in interviews | Alcohol |  |  |  |  |
|  |  |  |  | added based on triggers mentioned in interviews | Smoking (being near others who smoke, or smoking) |  |  |  |  |
|  |  |  |  | added based on triggers mentioned in interviews | Exercise |  |  |  |  |
|  |  |  |  | added based on triggers mentioned in interviews | Changes in altitude |  |  |  |  |
|  |  | Participants brought up symptoms outside of episodes during interviews, and study clinicians hypothesized that consistent experiences outside of episodes may help differentiate sinusitis from NRFP | Which of the following   symptoms do you experience outside of your episodes? (check one response per row) | Response options did not capture participants who experienced symptoms chronically | Responses choices modified to always or most of the time **outside of episodes**; sometimes **outside of episodes**; I do not experience this symptom outside of episode; **I experience this symptom chronically** |  |  |  |  |
|  |  |  | congestion |  |  |  |  |  |  |
|  |  |  | headache |  |  |  |  |  |  |
|  |  |  | sensitivity to sounds |  |  |  |  |  |  |
|  |  |  | Sensitivity to light |  |  |  |  |  |  |
|  |  |  | Watery or teary eyes |  |  |  |  |  |  |
|  |  |  | Blurry or hazy vision |  |  |  |  |  |  |
|  |  |  | Dizziness |  |  |  |  |  |  |
|  |  |  | Brain fog |  |  |  |  |  |  |
|  |  |  | Fatigue |  |  |  |  |  |  |
|  |  |  |  | participant brought this up in an interview | ear fullness |  |  |  |  |
