# Supplementary material for: Development of the Sinus Headache Screener to identify patients with non-rhinogenic facial pain compared with chronic rhinosinusitis in rhinology clinics
Source: J Patient Rep Outcomes. 2025 Nov 6;9:130. doi: 10.1186/s41687-025-00956-4 (PMC12592570; doi:10.1186/s41687-025-00956-4)
Supplement: Supplementary file 4 — Supplementary Material 4 [file 41687_2025_956_MOESM4_ESM.docx]

**Appendix 4. Number of participants in each round by diagnosis and overall n%**

|  | **NRFP (n = 15)** | **CRS (n = 11)** | **Overall (n = 26)** |
| --- | --- | --- | --- |
| **Round 1** | 5 (19.2%) | 4 (15.4%) | 9 (34.6%) |
| **Round 2** | 5 (19.2%) | 4 (15.4%) | 9 (34.6%) |
| **Round 3** | 5 (19.2%) | 3 (11.5%) | 8 (30.8%) |
