# Supplementary material for: Development of the Sinus Headache Screener to identify patients with non-rhinogenic facial pain compared with chronic rhinosinusitis in rhinology clinics
Source: J Patient Rep Outcomes. 2025 Nov 6;9:130. doi: 10.1186/s41687-025-00956-4 (PMC12592570; doi:10.1186/s41687-025-00956-4)
Supplement: Supplementary file 5 — Supplementary Material 5 [file 41687_2025_956_MOESM5_ESM.docx]

**Appendix 6. Frequency of Symptoms by Diagnosis**

| **Symptoms** | **NRFP** | **CRS** | **Overall** |
| --- | --- | --- | --- |
| nausea or feeling of being sick to your stomach | 10 (66.7%) | 3 (27.3%) | 50.0 |
| vomiting | 2 (13.3%) | 2 (18.2%) | 15.4 |
| trouble breathing through your nose | 8 (53.3%) | 10 (90.9%) | 69.2 |
| dizziness or trouble with balance | 12 (80.0%) | 6 (54.5%) | 69.2 |
| post-nasal drip | 11 (73.3%) | 10 (90.9%) | 80.8 |
| runny nose | 7 (46.7%) | 10 (90.9%) | 65.4 |
| stuffy nose | 10 (66.7%) | 11 (100.0%) | 80.8 |
| bright flashes of light that no one else saw | 4 (26.7%) | 2 (18.2%) | 23.1 |
| an experience of smelling odors that no one else smells | 6 (40.0%) | 2 (18.2%) | 30.8 |
| numbness in your face | 7 (46.7%) | 3 (27.3%) | 38.5 |
| tingling in your face | 6 (40.0%) | 3 (27.3%) | 34.6 |
| ringing in one or both ears | 33.3 | 4 (36.4%) | 34.6 |
| ear pain | 4 (26.7%) | 6 (54.5%) | 38.5 |
| ear fullness | 7 (46.7%) | 8 (72.7%) | 57.7 |
| headache | 13 (86.7%) | 11 (100.0%) | 92.3 |
| teary or watery eyes | 5 (33.3%) | 6 (54.5%) | 42.3 |
| an experience of light touch being painful | 9 (60.0%) | 4 (36.4%) | 50.0 |
| pain when chewing | 7 (46.7%) | 2 (18.2%) | 34.6 |
| light sensitivity | 12 (80.0%) | 5 (45.5%) | 65.4 |
| noise sensitivity | 10 (66.7%) | 2 (18.2%) | 46.2 |
| sore throat | 7 (46.7%) | 7 (63.6%) | 53.8 |
| green, yellow, or brown nasal discharge from nose | 7 (46.7%) | 9 (81.8%) | 61.5 |
| cough | 7 (46.7%) | 10 (90.9%) | 65.4 |
| tooth pain | 11 (73.3%) | 4 (36.4%) | 57.7 |
| sensitivity to smells | 8 (53.3%) | 2 (18.2%) | 38.5 |
| sensitivity to taste | 3 (20.0%) | 0 (0.0%) | 11.5 |
| foul taste in mouth | 6 (40.0%) | 5 (45.5%) | 42.3 |
| felt nauseated when reading in a moving vehicle | 4 (40.0%) | 4 (57.1%) | 47.1 |
| ear popping | 3 (30.0%) | 7 (100.0%) | 58.8 |
| twitching on face | 4 (40.0%) | 3 (42.9%) | 41.2 |
| brain fog | 3 (30.0%) | 4 (57.1%) | 70.6 |
| dry eyes | 4 (40.0%) | 5 (71.4%) | 52.9 |
| loss of vision | 2 (20.0%) | 3 (42.9%) | 29.4 |
| feeling of fluid in ears or head | 4 (40.0%) | 6 (85.7%) | 58.8 |
| inflammation under eyes | 2 (20.0%) | 1 (14.3%) | 17.6 |
| trouble Hearing | 1 (10.0%) | 1 (14.3%) | 11.8 |
| ear Infection | 0 (0.0%) | 1 (14.3%) | 5.9 |
| Loss of taste | 0 (0.0%) | 0 (0.0%) | 0.0 |
| Loss of ability to smell | 0 (0.0%) | 1 (14.3%) | 5.9 |
| Blurry/hazy vision | 3 (30.0%) | 3 (42.9%) | 35.3 |
| fatigue | 5 (50.0%) | 2 (28.6%) | 41.2 |
| pain on one side of your face | 3 (30.0%) | 1 (14.3%) | 23.5 |
| pain on both sides of your face | 4 (40.0%) | 1 (14.3%) | 29.4 |

Abbreviations: CRS=chronic rhinosinusitis; NRFP=non-rhinogenic facial pain
