# Supplementary material for: Development of the Sinus Headache Screener to identify patients with non-rhinogenic facial pain compared with chronic rhinosinusitis in rhinology clinics
Source: J Patient Rep Outcomes. 2025 Nov 6;9:130. doi: 10.1186/s41687-025-00956-4 (PMC12592570; doi:10.1186/s41687-025-00956-4)
Supplement: Supplementary file 6 — Supplementary Material 6 [file 41687_2025_956_MOESM6_ESM.docx]

**Appendix 7. Family and childhood history of migraine-related symptoms**

|  | **NRFP (n=15)** | **CRS (n=11)** | **Overall (n=26)** |
| --- | --- | --- | --- |
| **Family history of migraines** | 3 (20.0%) | 1 (9.1%) | 4 (15.4%) |
| **History of headaches as a child** | 4 (26.7%) | 1 (9.1%) | 5 (19.2%) |
| **History of motion sickness as a child** | 5 (26.7%) | 4 (36.4%) | 9 (34.6%) |

Abbreviations: CRS=chronic rhinosinusitis; NRFP=non-rhinogenic facial pain
