# Supplementary material for: Development of the Sinus Headache Screener to identify patients with non-rhinogenic facial pain compared with chronic rhinosinusitis in rhinology clinics
Source: J Patient Rep Outcomes. 2025 Nov 6;9:130. doi: 10.1186/s41687-025-00956-4 (PMC12592570; doi:10.1186/s41687-025-00956-4)
Supplement: Supplementary file 7 — Supplementary Material 7 [file 41687_2025_956_MOESM7_ESM.docx]

**Sinus Headache Screener (SHS) Item List**

**Patient screener – Step 1**

1. What are the reasons you are seeing your doctor today? (Please check all that apply.)

- Pain, discomfort or pressure in your face, neck, head (including eyes, ears, cheeks, eyebrows, jaw, temples, nose, or teeth)
- Congestion
- Runny nose
- Problems with sense of smell
- None of these
- Something else not listed, please specify: ________________

*[If answer is yes, continue to step 2. If answer is no, end screener.]*

**Patient screener – Step 2**

*Instructions:* We’d like to ask you about your symptoms so that we can provide the best care for you. You may or may not have experienced all the symptoms we will ask about, and that is OK. Please complete the following table of questions based on a **typical episode** of the condition you are seeking care for. If your episodes are chronic, please think about your chronic symptoms when answering these questions. Please do not answer about side effects you may experience due to medications for your condition. Your responses will help us determine next steps in your care.

***Section A - Symptoms***

1. During a **typical episode** do you experience … (Please check one response per row.)

|  |  | **Always** | **Very often** | **Sometimes** | **Rarely** | **Never** | **I don’t know** |
| --- | --- | --- | --- | --- | --- | --- | --- |
| A | Nausea or feeling of being sick to your stomach |  |  |  |  |  |  |
| B | Vomiting |  |  |  |  |  |  |
| C | Dizziness |  |  |  |  |  |  |
| D | Trouble with balance |  |  |  |  |  |  |
| E | Lightheadedness |  |  |  |  |  |  |
| F | Felt nauseated when reading in a moving vehicle |  |  |  |  |  |  |
| G | Motion sickness |  |  |  |  |  |  |
| H | Trouble breathing through your nose |  |  |  |  |  |  |
| I | Post-nasal drip |  |  |  |  |  |  |
| J | Runny nose |  |  |  |  |  |  |
| K | Stuffy nose |  |  |  |  |  |  |
| L | Green, yellow, or brown nasal discharge from nose |  |  |  |  |  |  |
| M | Cough |  |  |  |  |  |  |
| N | Bright flashes of light that no one else saw |  |  |  |  |  |  |
| O | Light sensitivity |  |  |  |  |  |  |
| P | Noise sensitivity |  |  |  |  |  |  |
| Q | Trouble hearing |  |  |  |  |  |  |
| R | Loss of taste |  |  |  |  |  |  |
| S | Loss of ability to smell |  |  |  |  |  |  |
| T | An experience of smelling odors that no one else smells |  |  |  |  |  |  |
| U | Sensitivity to smells |  |  |  |  |  |  |
| V | Sensitivity to taste |  |  |  |  |  |  |
| W | Loss of vision |  |  |  |  |  |  |
| X | Numbness in your face |  |  |  |  |  |  |
| Y | Pain on one side of your face |  |  |  |  |  |  |
| Z | Pain on both sides of your face |  |  |  |  |  |  |
| AA | An experience of light touch being painful |  |  |  |  |  |  |
| AB | Tingling in your face |  |  |  |  |  |  |
| AC | Twitching on face |  |  |  |  |  |  |
| AD | Blurry/hazy vision |  |  |  |  |  |  |
| AE | Ringing in one or both ears |  |  |  |  |  |  |
| AF | Ear pain |  |  |  |  |  |  |
| AG | Ear fullness |  |  |  |  |  |  |
| AH | Ear popping |  |  |  |  |  |  |
| AI | Feeling of fluid in ears or head |  |  |  |  |  |  |
| AJ | Ear infection |  |  |  |  |  |  |
| AK | Headache |  |  |  |  |  |  |
| AL | Teary or watery eyes |  |  |  |  |  |  |
| AM | Dry eyes |  |  |  |  |  |  |
| AN | Inflammation or swelling under eyes |  |  |  |  |  |  |
| AO | Pain when chewing |  |  |  |  |  |  |
| AP | Tooth pain or sensitivity |  |  |  |  |  |  |
| AQ | Throat discomfort |  |  |  |  |  |  |
| AR | Foul taste in mouth |  |  |  |  |  |  |
| AS | Brain fog |  |  |  |  |  |  |
| AT | Fatigue |  |  |  |  |  |  |

2XX. If you experience symptoms that were not listed here, please let us know what those are: _________________________________________________________________________

***Section B - Episode Characteristics***

1. How long do your **typical episodes** usually last?

- Less than 24 hours
- 1-3 days
- 4-7 days
- 8-14 days
- More than 2 weeks
- Chronic symptoms (daily or almost daily)

1. During your **typical episodes**, how would you describe your mucus/snot? (Please check all that apply.)

- Thick mucus
- Thin mucus
- Cloudy mucus
- Clear mucus
- Yellow, green, or brown mucus
- I do not have mucus or runny nose during my episodes.
- I am unable to determine what my mucus/snot looks like during my episodes.

***Section C - Triggers***

1. [Skip if #3 = “Chronic symptoms”] Do any of the following regularly trigger an episode? (check one response per row)

|  |  | **Yes** | **No** |
| --- | --- | --- | --- |
| A | Stress |  |  |
| B | Poor sleep |  |  |
| C | Smells |  |  |
| D | Allergies (seasonal, pets) |  |  |
| E | Changes in pressure due to weather |  |  |
| F | Having a cold |  |  |
| G | Humidity |  |  |
| H | Menstrual cycle |  |  |
| I | Alcohol |  |  |
| J | Smoking (being near others who smoke, or smoking) |  |  |
| K | Exercise |  |  |
| L | Changes in altitude |  |  |

5XX. If you experience triggers to your episodes that are not listed in the table above, please list them here: ____________________________________________________________________________

***Section D - Symptoms outside of episodes***

1. [Skip if #3 = “Chronic symptoms”] Which of the following symptoms do you experience outside of your episodes? (check one response per row.)

|  |  | **Always or most of the time outside of episodes** | **Sometimes outside of episodes** | **I do not experience this symptom outside of episodes** | **I experience this symptom chronically** |
| --- | --- | --- | --- | --- | --- |
| A | Congestion |  |  |  |  |
| B | Headache |  |  |  |  |
| C | Sensitivity to sounds |  |  |  |  |
| D | Sensitivity to light |  |  |  |  |
| E | Watery or teary eyes |  |  |  |  |
| F | Blurry or hazy vision |  |  |  |  |
| G | Dizziness |  |  |  |  |
| H | Brain fog |  |  |  |  |
| I | Fatigue |  |  |  |  |
| J | Ear fullness |  |  |  |  |

6XX. If you experience any symptoms regularly outside of your episodes that were not listed in the table above, please indicate them here: ____________________________________________________________________________

***Section E - Treatment***

1. Do any of the following treatments help to address some or all of your symptoms during **typical episodes**? (Check one response per row.)

|  |  | **Always** | **Very often** | **Sometimes** | **Rarely** | **Never** | **I have not tried these** |
| --- | --- | --- | --- | --- | --- | --- | --- |
| A | Nasal steroid sprays (such as Flonase, Nasonex) |  |  |  |  |  |  |
| B | Antihistamines (such as Benadryl, Claritin, Zyrtec, Allegra) |  |  |  |  |  |  |
| C | Migraine medications (such as Imitrex/sumatriptan) |  |  |  |  |  |  |
| D | Antibiotics |  |  |  |  |  |  |
| E | Oral steroids |  |  |  |  |  |  |
| F | Caffeine |  |  |  |  |  |  |
| G | Over-the-counter pain reliever (such as Advil, Tylenol) |  |  |  |  |  |  |
| H | Decongestants (such as Sudafed, Claritin-D, Allegra-D, Afrin) |  |  |  |  |  |  |
| I | Injectable medications (such as Dupixant) |  |  |  |  |  |  |
| J | Nasal saline rinse |  |  |  |  |  |  |

***Section F - History***

1. Do you have any family history of migraine or unexplained headaches?
   - Yes
   - No
   - I do not know
2. Do you recall having unexplained headaches as a child?
   - Yes
   - No
   - I do not know
3. Do you recall having motion sickness as a child?
   - Yes
   - No
   - I do not know

***Section G - Severity***

1. How would you rate the level of discomfort you feel on your face, neck, or head (including eyes, ears, cheeks, eyebrows, jaw, temples, or nose) during a **typical episode**?
   - 1 = no discomfort
   - 2
   - 3
   - 4
   - 5 = worst discomfort
2. To what extent do your **typical episodes** limit your activities?
   - Not at all
   - Very little
   - Somewhat
   - Quite a lot
   - Completely - I cannot conduct my activities
