# Supplementary material for: Development of the Sinus Headache Screener to identify patients with non-rhinogenic facial pain compared with chronic rhinosinusitis in rhinology clinics
Source: J Patient Rep Outcomes. 2025 Nov 6;9:130. doi: 10.1186/s41687-025-00956-4 (PMC12592570; doi:10.1186/s41687-025-00956-4)
Supplement: Supplementary file 8 — Supplementary Material 8 [file 41687_2025_956_MOESM8_ESM.docx]

**Appendix 2. Concept Elicitation Interview Guide Questions (Rounds 1-3)**

**Interview Guide: Round 1 (2022-03-07)**

*Date of interview: ___________________*

*Participant ID number: __________________*

*Interviewer’s name: __________________*

*Patient diagnosis: ____________________*

*Interview Start Time (24-hour clock): ______: ______*

*Interview Setting:*

| *□ Virtual* | *□ Telephone* |
| --- | --- |

**SECTION A: Read Introduction Page to Participant**

My name is xxx from Duke’s Department of Population Health Sciences. We greatly appreciate your making time to talk to us today.

As a reminder, the goal of this study is to learn about your experience with discomfort you may have in your face, head, or neck. We will go over some draft survey questions about symptoms associated with your condition, and ask for your feedback on those survey questions.

The interview will take about an hour. Your participation is completely voluntary. You can choose to take a break at any time or stop the interview entirely.

There are no right or wrong answers. We are interested in your thoughts and experiences.

I would like to audio record the interview because I want to make sure I don’t miss any of your comments. If you don’t want the interview audio recorded, I will take detailed notes during the interview instead. As a reminder, the recording will be stored securely and destroyed after we publish the study’s findings. We will not associate your name with your responses.

[*If interview is virtual:* I am going to go ahead and turn off the video at this time too.]

Do I have your permission to record this interview?

□Yes [Begin interview recording.] □No

Do you have any questions for me so far about the interview?

## Section B: Concept Elicitation

In the interview today, I’d like to start by asking you about what I refer to as “xxx”. By xxx, what I mean is the condition you saw the doctor for related to the discomfort you experienced in your face, head or neck.

1. Does the term, xxx, seem fitting to you for what I described?
   1. *[If no]* Is there a different phrase that you would use? [*Interviewer: if the patient offers a different term that is a synonym for* xxx*, use that term throughout the interview.]*
   2. *[Interviewer: If the term seems different from* xxx*]* Tell me what [*patient’s term*] means to you. Could you define it for me? *[Interviewer: If the term seems like a synonym]* Which term would you like to use during the interview today?

*[If the term does not seem like a synonym of* xxx*, agree upon a term that the patient understands as* xxx*.* xxx *has been blue-italicized throughout the interview guide to remind you when to use the patients’ wording.]*

1. Do you experience pain, pressure, or discomfort anywhere on your face, neck, or head (including eyes, ears, cheeks, temples, or nose) during your xxx episodes?
   1. Please describe your experience with discomfort to me in your own words.
      1. Probe on:
         1. location of discomfort
         2. what terms they use for the discomfort (pain, pressure, tingling, numbness, fullness, something else)
            1. If pain – How would you describe the pain – throbbing, shooting, fullness, pressure, anything else?
   2. *[If no]* Do you experience any other type of symptoms related to your head, neck, or face?

1. Please tell me about your most recent experience with xxx. What other symptoms did you experience?
2. Please tell me about your experience with xxx when the episode has been the most severe.
   1. Was your most recent episode your most severe episode?
   2. [If most severe is the most recent, then skip this question] How is your most severe episode different from your most recent episode?
   3. How is your most-severe episode different from a typical episode?
3. During a typical episode, do you experience the same constellation of symptoms throughout an episode, or do the symptoms you have change throughout an episode?
4. How long have your episodes lasted (hours, days, weeks, months)?
   1. Probe: How do you know when your episodes are starting? Ending?
5. Have you experienced a runny nose or congestion with your episodes?
   1. *[If yes]* Could you tell me more about your mucus (snot) with your runny nose or congestion? What color was it (yellow, brown, red, clear)? Was the consistency thick or thin? Was the mucus/snot cloudy? Can you think of anything else that was different about your mucus/snot with your episode from other times when you’ve had a runny nose or congestion?
6. Have you been diagnosed with any other conditions that sometimes feel like xxx? If yes, what conditions are they? What do they have in common with your episodes? How do you know if your symptoms are due to an episode versus the other condition(s)?
7. What do you do to help yourself feel better during an episode?
   1. Are there any medications or treatments that help the symptoms you experience with your episodes? (Probe as necessary: “Triptan”, Amitriptyline, topiramate, propranolol, Sumatriptan, Imitrex, Sudafed, Tylenol, NSAIDS, Mucinex, Flonase, antihistamine, antibiotics, prednisone)
   2. Are there any medications or treatments you have tried that do not work?
8. What triggers your episodes, if anything?
   1. Probes: weather, seasons, altitude changes, travel, screen time, physical activity, stress, chewing, poor sleep, bright lights, smells, eating or drinking something in particular
   2. Does the episode begin every time after you have this/these triggers? Have you had any episodes without a trigger?
9. During an episode, what can make your symptoms worse?
   1. Probes: weather, seasons, altitude changes, travel, screen time, physical activity, stress, chewing, poor sleep, bright lights, smells, eating or drinking something in particular
10. Now I’d like to talk about some things that might happen outside of your episodes. Do you experience any less-severe symptoms throughout the week when you are not having an episode?
    1. Probe: headache, light sensitivity, sensitivity to sounds, “brain fog”, dizziness, fatigue or blurry vision, congestion, migraines
    2. How often do you experience these throughout the week?
11. Are you aware of any family history of migraine? [Probe on immediate family members that have migraine.]
12. As a child, did you experience any unexplained headaches, stomach aches, or dizziness for unknown reasons? motion sickness?

## Section C: Cognitive Interview

Now we are going to switch gears and look at some survey questions about *[xxx]*. Do you have the survey questions with you? Some of the questions may seem repetitive, but they are asking about slightly different things.

1. Let’s begin by taking a look at question **1**. For this question, let’s pretend you are at your doctor’s office. Please read the question to yourself.
   1. What response did you choose for this question? [Probe if needed:] How did you get to your answer?
   2. Is there a different term that you would use to describe your experience other than “discomfort”? [Probe if needed: Of the terms you mentioned, is there one that resonates with you the most?]
2. Let’s move on to question **2**. Please read the instructions to yourself. What are the instructions asking you to do?

[*If patient did not totally understand the instructions, explain what we’d like them to do and ask:*] Is there anything we can do to make the instructions more clear?

1. Now we will go through each of the questions in the table together. We will use a think aloud technique. Please read the question to yourself. Then, tell me what you chose for each experience, and how you got to your answer. [Interviewer: record responses and notes in the table.]

During a **typical episode** do you have… (check one response per row) [Reminder: Record patient responses]

|  |  | **Yes** | **No** | **I don’t know** | **Interviewer notes** |
| --- | --- | --- | --- | --- | --- |
| a | nausea or feeling of being sick to your stomach |  |  |  |  |
| b | vomiting |  |  |  |  |
| c | trouble breathing through your nose |  |  |  |  |
| d | dizziness or trouble with balance |  |  |  |  |
| e | post-nasal drip |  |  |  |  |
| f | new or worsening runny nose |  |  |  |  |
| g | new or worsening stuffy nose |  |  |  |  |
| h | bright flashes of light that no one else saw |  |  |  |  |
| i | an experience of smelling odors that no one else smells |  |  |  |  |
| j | numbness in your face |  |  |  |  |
| k | tingling in your face |  |  |  |  |
| l | ringing in one or both ears |  |  |  |  |
| m | ear pain |  |  |  |  |
| n | ear fullness |  |  |  |  |
| o | headache |  |  |  |  |
| p | teary or watery eyes |  |  |  |  |
| q | an experience of light touch being painful |  |  |  |  |
| r | pain when chewing |  |  |  |  |
| S | light sensitivity |  |  |  |  |
| T | noise sensitivity |  |  |  |  |
| U | sore throat |  |  |  |  |
| V | green, yellow, or brown nasal discharge from nose |  |  |  |  |
| W | cough |  |  |  |  |
| X | tooth pain |  |  |  |  |
| Y | sensitivity to smells |  |  |  |  |
| z | sensitivity to taste |  |  |  |  |
| aa | foul taste in mouth |  |  |  |  |

- 1. How easy or difficult was it to think back to your “typical episode”?
  2. [Probe if needed:] What made it easy? What made it difficult?
  3. How easy or difficult was it to answer questions in this format?
     1. Probe: What made it difficult? What made it easy?
     2. [Probe if necessary:] What did you like or not like about the table format?

1. Now let’s look at Section B. Please start with question 3. Please read the question to yourself. What was your answer to this question? How did you choose your response? [*If a patient chooses a response choice that seems different from their experience, probe further by asking about the other response choices and what their experience would be like if they were to choose the response you thought they might choose.*]
2. Let’s look at Section C. Please read and answer the questions to yourself. Please circle or jot down issues you see, or things that do not make sense. Then, we will talk about this group of questions together.
   - 1. What were your answers for these questions?

4

5

6

7

8

- - 1. Did you have any trouble answering the questions in this section? Were any questions unclear?
    2. Did you answer “I do not know” for any of these questions? How did you get to that answer?

1. Is there anything about your experience with *xxx* that we did not talk about that you think would be important for us to know?

Thank you very much for your help today.

Before we end the interview, I have a short demographics form for you to complete. The answers to these questions help us describe the people who participated in the study. We will report a summary of these data only.

Do you have any other questions? Thank you again. [we will mail you a check], as thanks for participating in this study.

**Interview Guide: Round 2 (2022-04-13)**

*Date of interview: ___________________*

*Participant ID number: __________________*

*Interviewer’s name: __________________*

*Patient diagnosis: ____________________*

*Interview Start Time (24-hour clock): ______: ______*

*Interview Setting:*

| *□ Virtual* | *□ Telephone* |
| --- | --- |

**SECTION A: Read Introduction Page to Participant**

My name is xxx from Duke’s Department of Population Health Sciences. We greatly appreciate your making time to talk to us today.

As a reminder, the goal of this study is to learn about your experience with discomfort you may have in your face, head, or neck. We will go over some draft survey questions about symptoms associated with your condition, and ask for your feedback on those survey questions.

The interview will take about an hour. Your participation is completely voluntary. You can choose to take a break at any time or stop the interview entirely.

There are no right or wrong answers. We are interested in your thoughts and experiences. I also want to assure you that I am in a private room and no one else can hear your responses during the interview.

As a reminder, you will receive a $25 check for participating today. We will need to collect your social security number and mailing address in order to process the payment. We collect this information because we are required by the IRS to report how much people receive from participating in research at Duke. If you are not open to sharing this information with me, please let me know. We can still conduct the interview, but we will not be able to compensate you for your time.

I would also like to audio record the interview because I want to make sure I don’t miss any of your comments. I will only keep the audio portion of the recording; the video version will be permanently deleted after the interview is completed. If you don’t want the interview audio recorded, I will take detailed notes during the interview instead. As a reminder, the recording will be stored securely and destroyed after we publish the study’s findings. We will not associate your name with your responses.

[*If interview is virtual:* I am going to go ahead and turn off the video at this time too.]

Do I have your permission to record this interview?

□Yes [Begin interview recording.] □No

Do you have any questions for me so far about the interview?

## Section B: Concept Elicitation

In the interview today, I’d like to start by asking you about what I refer to as “xxx”. By xxx, what I mean is the condition you saw the doctor for related to the discomfort you experienced in your face, head or neck.

1. Does the term, xxx, seem fitting to you for what I described?
   1. *[If no]* Is there a different phrase that you would use? [*Interviewer: if the patient offers a different term that is a synonym for* xxx*, use that term throughout the interview.]*
   2. *[Interviewer: If the term seems different from* xxx*]* Tell me what [*patient’s term*] means to you. Could you define it for me? *[Interviewer: If the term seems like a synonym]* Which term would you like to use during the interview today?

*[If the term does not seem like a synonym of* xxx*, agree upon a term that the patient understands as* xxx*.* xxx *has been blue-italicized throughout the interview guide to remind you when to use the patients’ wording.]*

1. Do you experience pain, pressure, or discomfort anywhere on your face, neck, or head (including eyes, ears, cheeks, temples, or nose) during your xxx episodes?
   1. Please describe your experience with discomfort to me in your own words.
      1. Probe on:
         1. location of discomfort
         2. what terms they use for the discomfort (pain, pressure, tingling, numbness, fullness, something else)
            1. If pain – How would you describe the pain – throbbing, shooting, fullness, pressure, anything else?
   2. Do you experience any other type of symptoms related to your head, neck, or face?
2. How would you describe a typical episode? What makes an episode typical compared to other types of episodes (most severe, least severe, most recent)? How do you describe other types of episodes?
3. How long do your typical episodes last (hours, days, weeks, months)?
   1. Probe: How do you know when your episodes are starting? Ending?
4. Have you been diagnosed with any other conditions that sometimes feel like xxx? If yes, what conditions are they? What do they have in common with your episodes? How do you know if your symptoms are due to an episode versus the other condition(s)?

## Section C: Cognitive Interview

Now we are going to switch gears and look at some survey questions about *[xxx]*. Do you have the survey questions with you? Some of the questions may seem repetitive, but they are asking about slightly different things.

1. Let’s begin by taking a look at question **1**. For this question, let’s pretend you are at your doctor’s office. Please read the question to yourself.
   1. What response did you choose for this question? [Probe if needed:] How did you get to your answer?
   2. Is there a different term that you would use to describe your experience other than “discomfort”? [Probe if needed: Of the terms you mentioned, is there one that resonates with you the most?]
2. Let’s move on to question **2**. Please read the instructions to yourself. What are the instructions asking you to do?

[*If patient did not totally understand the instructions, explain what we’d like them to do and ask:*] Is there anything we can do to make the instructions more clear?

1. Now we will go through each of the questions in the table together. We will use a think aloud technique. Please read the question to yourself. Then, tell me what you chose for each experience, and how you got to your answer. [Interviewer: record responses and notes in the table.]

During a **typical episode** do you experience… (check one response per row) [Reminder: Record patient responses; note any responses that surprised you due to inconsistencies]

|  |  | **Always or most of the time** | **Sometimes** | **Never** | **I don’t know** |
| --- | --- | --- | --- | --- | --- |
| A | nausea or feeling of being sick to your stomach |  |  |  |  |
| B | vomiting |  |  |  |  |
| C | trouble breathing through your nose |  |  |  |  |
| D | dizziness or trouble with balance |  |  |  |  |
| E | post-nasal drip |  |  |  |  |
| F | runny nose |  |  |  |  |
| G | stuffy nose |  |  |  |  |
| H | bright flashes of light that no one else saw |  |  |  |  |
| I | an experience of smelling odors that no one else smells |  |  |  |  |
| J | numbness in your face |  |  |  |  |
| K | tingling in your face |  |  |  |  |
| L | ringing in one or both ears |  |  |  |  |
| M | ear pain |  |  |  |  |
| N | ear fullness |  |  |  |  |
| O | headache |  |  |  |  |
| P | teary or watery eyes |  |  |  |  |
| Q | an experience of light touch being painful |  |  |  |  |
| R | pain when chewing |  |  |  |  |
| S | light sensitivity |  |  |  |  |
| T | noise sensitivity |  |  |  |  |
| U | sore throat |  |  |  |  |
| V | green, yellow, or brown nasal discharge from nose |  |  |  |  |
| W | cough |  |  |  |  |
| X | tooth pain |  |  |  |  |
| Y | sensitivity to smells |  |  |  |  |
| Z | sensitivity to taste |  |  |  |  |
| AA | foul taste in mouth |  |  |  |  |
| AB | nausea when reading in a moving vehicle |  |  |  |  |
| AC | ear popping |  |  |  |  |
| AD | twitching on face |  |  |  |  |
| AE | brain fog |  |  |  |  |
| AF | dry eyes |  |  |  |  |
| AG | loss of vision |  |  |  |  |
| AH | feeling of fluid in ears or head |  |  |  |  |
| AI | inflammation under eyes |  |  |  |  |

- 1. How easy or difficult was it to think back to your “typical episodes”?
  2. [Probe if needed:] What made it easy? What made it difficult?
  3. Is there anything missing from this table that you experience during your typical episodes? [Probe if needed:] If yes, tell me about those things.

1. Let’s move on to question **3**. Please read the question to yourself. What answer did you choose? How did you decide this was the best answer for you?

[interviewer: if participant chooses more than two weeks, ask them how long they typically last.]

1. Let’s look at question **4**. Please read the question to yourself. What response or responses did you choose? How did you choose the responses?

[interviewer: note any responses that surprise you due to inconsistencies.]

1. Let’s look at question **5**. What is the question asking you to do? [Interviewer: if participant gets it wrong, explain the intent of the question and then ask how we could make the intent clearer.]
   1. Let’s go through each row in question 5. We will use the think aloud technique again. Please choose a response for each row and tell me how you got to your answer. [Reminder: Record patient responses]

Do any of the following regularly trigger an episode? (check one response per row)

|  |  | **Yes** | **No** |
| --- | --- | --- | --- |
| A | Stress |  |  |
| B | Poor sleep |  |  |
| C | Smells |  |  |
| D | Allergies (seasonal, pets) |  |  |
| E | Changes in barometric pressure |  |  |
| F | Having a cold |  |  |

- 1. Is there anything missing from this table? [Probe if needed:] If yes, tell me about those things.

1. Let’s move on to question **6**. What is this question asking you to do? [Interviewer: if participant gets it wrong, explain the intent of the question and then ask how we could make the intent clearer.]
   1. Let’s go through each row in question 6 using the think aloud technique again. Please choose a response for each row and tell me how you got to your answer. [Reminder: Record participant responses]

Which of the following symptoms do you experience outside of your episodes? (check one response per row)

|  |  | Always or most of the time | Sometimes | I do not experience this symptom outside of episodes |
| --- | --- | --- | --- | --- |
| A | Congestion |  |  |  |
| B | Headache |  |  |  |
| C | Sensitivity to sounds |  |  |  |
| E | Sensitivity to light |  |  |  |
| E | Watery or teary eyes |  |  |  |
| F | Blurry or hazy vision |  |  |  |
| G | Dizziness |  |  |  |
| H | Brain fog |  |  |  |
| I | Fatigue |  |  |  |

- 1. Do you experience any symptoms between episodes that are missing from this table? [Probe if needed:] If yes, please tell me about those things.

1. Let’s move on to question **7**. What is this question asking you to do? [Interviewer: if participant gets it wrong, explain the intent of the question and then ask how we could make the intent clearer.]
   1. Let’s go through each row in question 7 with the think aloud technique. Please choose a response for each row and please tell me how you got to your answer. [Reminder: Record participant responses]

Do any of the following treatments help to address symptoms during your typical episodes? (check one response per row)

[Interviewer: Ask participant to clarify which specific medications helps them]

|  |  | Always or most of the time | Sometimes | No | I have not tried this treatment |
| --- | --- | --- | --- | --- | --- |
| A | Nasal sprays (such as Flonase) |  |  |  |  |
| B | Antihistamines (such as Benadryl, Claritin, Zyrtec, Allegra) |  |  |  |  |
| C | Migraine medications (such as Imitrex/sumatriptan) |  |  |  |  |
| D | Antibiotics |  |  |  |  |
| E | Oral steroids |  |  |  |  |
| F | Caffeine |  |  |  |  |
| G | Over-the-counter pain reliever (such as Advil, Tylenol) |  |  |  |  |

1. Let’s look at questions **8 through 10**. Please read these questions to yourself.
   1. What response did you choose for each question? [Reminder: Record participant responses below] How did you get to your response?

| Q8 – family history of migraine |  |
| --- | --- |
| Q9 – unexplained headaches as a child |  |
| Q10 – motion sickness as a child |  |

1. Only a few more questions left! Let’s look at question **11**. Please read the question to yourself. Please tell me what response you choose.
   1. How did you decide this was the best response for you?
   2. [Interviewer: choose a response choice 1 up or 1 down from the response choice and ask:] Please describe how you would feel differently if you chose response choice x.


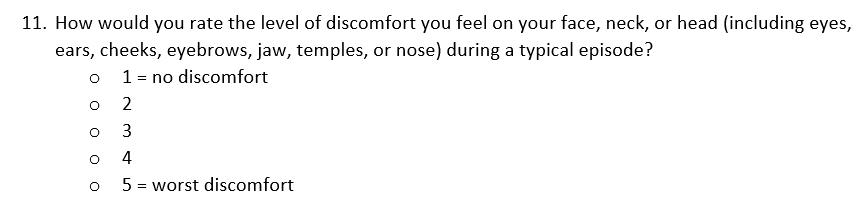


1. Looking at question **12** – please read the question to yourself. What response did you choose?
   1. How did you get to your response?
2. If there was a new medication available to improve your experiences with [xxx condition], what aspects of [xxx condition] are most important to you to be changed?
   1. [Probe:] Which symptoms?
   2. [Probe:]Which impacts? (social function, physical function, ability to work, ability to do hobbies)

Thank you very much for your help today.

Before we end the interview, I have a short demographics form for you to complete. The answers to these questions help us describe the people who participated in the study. We will report a summary of these data only.

Do you have any other questions? Thank you again. [we will mail you a check], as thanks for participating in this study.

**Interview Guide: Round 3 (2022-06-07)**

*Date of interview: ___________________*

*Participant ID number: __________________*

*Interviewer’s name: __________________*

*Patient diagnosis: ____________________*

*Interview Start Time (24-hour clock): ______: ______*

*Interview Setting:*

| *□ Virtual* | *□ Telephone* |
| --- | --- |

**SECTION A: Read Introduction Page to Participant**

My name is xxx from Duke’s Department of Population Health Sciences. We greatly appreciate your making time to talk to us today.

As a reminder, the goal of this study is to learn about your experience with discomfort you may have in your face, head, or neck. We will go over some draft survey questions about symptoms associated with your condition, and ask for your feedback on those survey questions.

The interview will take about an hour. Your participation is completely voluntary. You can choose to take a break at any time or stop the interview entirely.

There are no right or wrong answers. We are interested in your thoughts and experiences. I also want to assure you that I am in a private room and no one else can hear your responses during the interview.

As a reminder, you will receive a $25 check for participating today. We will need to collect your social security number and mailing address in order to process the payment. We collect this information because we are required by the IRS to report how much people receive from participating in research at Duke. If you are not open to sharing this information with me, please let me know. We can still conduct the interview, but we will not be able to compensate you for your time.

I would also like to audio record the interview because I want to make sure I don’t miss any of your comments. I will only keep the audio portion of the recording; the video version will be permanently deleted after the interview is completed. If you don’t want the interview audio recorded, I will take detailed notes during the interview instead. As a reminder, the recording will be stored securely and destroyed after we publish the study’s findings. We will not associate your name with your responses.

[*If interview is virtual:* I am going to go ahead and turn off the video at this time too.]

Do I have your permission to record this interview?

□Yes [Begin interview recording.] □No

Do you have any questions for me so far about the interview?

## Section B: Concept Elicitation

In the interview today, I’d like to start by asking you about what I refer to as “xxx”. By xxx, what I mean is the condition you saw the doctor for related to the discomfort you experienced in your face, head or neck.

1. Does the term, xxx, seem fitting to you for what I described?
   1. *[If no]* Is there a different phrase that you would use? [*Interviewer: if the patient offers a different term that is a synonym for* xxx*, use that term throughout the interview.]*
   2. *[Interviewer: If the term seems different from* xxx*]* Tell me what [*patient’s term*] means to you. Could you define it for me? *[Interviewer: If the term seems like a synonym]* Which term would you like to use during the interview today?

*[If the term does not seem like a synonym of* xxx*, agree upon a term that the patient understands as* xxx*.* xxx *has been blue-italicized throughout the interview guide to remind you when to use the patients’ wording.]*

1. [If a sinusitis patient] Have you had surgery to address symptoms of sinusitis? What happened with your sinusitis symptoms after surgery?

[Reminder: If patients’ symptoms have been resolved, we still want them to go through the interview as they usually would letting us know how they’d answer questions if they took the screener today.]

1. Do you experience pain, pressure, or discomfort anywhere on your face, neck, or head (including eyes, ears, cheeks, temples, or nose) during your xxx episodes?
   1. Please describe your experience with discomfort to me in your own words.
      1. Probe on:
         1. location of discomfort
         2. what terms they use for the discomfort (pain, pressure, tingling, numbness, fullness, something else)
            1. If pain – How would you describe the pain – throbbing, shooting, fullness, pressure, anything else?
   2. Do you experience any other type of symptoms related to your head, neck, or face?
2. Do your symptoms come and go, or are they around most of the time?
   1. [If symptoms come and go] When you talk with your doctor about your symptoms, what term do you use to describe the times when you experience symptoms?

[Wait for response, and then offer suggestions for terms to see what resonates with patient: episode, event, flare-up, something else]

1. How would you describe a typical episode? What makes an episode typical compared to other types of episodes (most severe, least severe, most recent)? How do you describe other types of episodes?
2. How long do your typical episodes last (hours, days, weeks, months)?
   1. Probe: How do you know when your episodes are starting? Ending?
3. Have you been diagnosed with any other conditions that sometimes feel like xxx? If yes, what conditions are they? What do they have in common with your episodes? How do you know if your symptoms are due to an episode versus the other condition(s)?

## Section C: Cognitive Interview

Now we are going to switch gears and look at some survey questions about *[xxx]*. Do you have the survey questions with you? Some of the questions may seem repetitive, but they are asking about slightly different things.

1. Let’s begin by taking a look at question **1**. For this question, let’s pretend you are at your doctor’s office being seen for xxx. Please read the question to yourself.
   1. What response did you choose for this question? [Probe if needed:] How did you get to your answer?
   2. Is there a different term that you would use to describe your experience other than “discomfort”, “pain”, or “pressure”? [Probe if needed: Of the terms you mentioned, is there one that resonates with you the most?]
2. Let’s move on to question **2**. Please read the instructions to yourself. What are the instructions asking you to do? What does the term “episode” mean to you?

[*If patient did not totally understand the instructions, explain what we’d like them to do and ask:*] Is there anything we can do to make the instructions more clear?

1. Now we will go through each of the questions in the table together. We will use a think aloud technique. Please read the question to yourself. Then, tell me what you chose for each experience, and how you got to your answer. [Interviewer: record responses and notes in the table.]

During a **typical episode** do you experience… (check one response per row) [Reminder: Record patient responses; note any responses that surprised you due to inconsistencies]

|  |  | **Always** | **Very often** | **Sometimes** | **Rarely** | **Never** | **I don’t know** |
| --- | --- | --- | --- | --- | --- | --- | --- |
| A | nausea or feeling of being sick to your stomach |  |  |  |  |  |  |
| B | vomiting |  |  |  |  |  |  |
| C | trouble breathing through your nose |  |  |  |  |  |  |
| D | dizziness or trouble with balance |  |  |  |  |  |  |
| E | post-nasal drip |  |  |  |  |  |  |
| F | runny nose |  |  |  |  |  |  |
| G | stuffy nose |  |  |  |  |  |  |
| H | bright flashes of light that no one else saw |  |  |  |  |  |  |
| I | an experience of smelling odors that no one else smells |  |  |  |  |  |  |
| J | numbness in your face |  |  |  |  |  |  |
|  |  | **Always** | **Very often** | **Sometimes** | **Rarely** | **Never** | **I don’t know** |
| K | tingling in your face |  |  |  |  |  |  |
| L | ringing in one or both ears |  |  |  |  |  |  |
| M | ear pain |  |  |  |  |  |  |
| N | ear fullness |  |  |  |  |  |  |
| O | headache |  |  |  |  |  |  |
| P | teary or watery eyes |  |  |  |  |  |  |
| Q | an experience of light touch being painful |  |  |  |  |  |  |
| R | pain when chewing |  |  |  |  |  |  |
| S | light sensitivity |  |  |  |  |  |  |
| T | noise sensitivity |  |  |  |  |  |  |
| U | throat discomfort |  |  |  |  |  |  |
| V | green, yellow, or brown nasal discharge from nose |  |  |  |  |  |  |
| W | cough |  |  |  |  |  |  |
| X | tooth pain |  |  |  |  |  |  |
| Y | sensitivity to smells |  |  |  |  |  |  |
| Z | sensitivity to taste |  |  |  |  |  |  |
| AA | foul taste in mouth |  |  |  |  |  |  |
| AB | felt nauseated when reading in a moving vehicle |  |  |  |  |  |  |
| AC | ear popping |  |  |  |  |  |  |
| AD | twitching on face |  |  |  |  |  |  |
| AE | brain fog |  |  |  |  |  |  |
| AF | dry eyes |  |  |  |  |  |  |
| AG | loss of vision |  |  |  |  |  |  |
| AH | feeling of fluid in ears or head |  |  |  |  |  |  |
| AI | inflammation under eyes |  |  |  |  |  |  |
| AJ | trouble hearing |  |  |  |  |  |  |
| AK | ear infection |  |  |  |  |  |  |
| AL | loss of taste |  |  |  |  |  |  |
| AM | loss of ability to smell |  |  |  |  |  |  |
| AN | blurry/hazy vision |  |  |  |  |  |  |
| AO | fatigue |  |  |  |  |  |  |
| AP | pain on one side of your face |  |  |  |  |  |  |
| AQ | pain on both sides of your face |  |  |  |  |  |  |

- 1. How easy or difficult was it to think back to your “typical episodes”?
  2. [Probe if needed:] What made it easy? What made it difficult?
  3. Is there anything missing from this table that you experience during your typical episodes? [Probe if needed:] If yes, tell me about those things.

1. Let’s move on to question **3**. Please read the question to yourself. What answer did you choose? How did you decide this was the best answer for you?

[interviewer: if participant chooses more than two weeks, ask them how long they typically last.]

1. Let’s look at question **4**. Please read the question to yourself. What response or responses did you choose? How did you choose the responses?

[interviewer: note any responses that surprise you due to inconsistencies.]

1. Let’s look at question **5**. What is the question asking you to do? [Interviewer: if participant gets it wrong, explain the intent of the question and then ask how we could make the intent clearer.]
   1. Let’s go through each row in question 5. We will use the think aloud technique again. Please choose a response for each row and tell me how you got to your answer. [Reminder: Record patient responses]

Do any of the following regularly trigger an episode? (check one response per row)

|  |  | **Yes** | **No** |
| --- | --- | --- | --- |
| A | Stress |  |  |
| B | Poor sleep |  |  |
| C | Smells |  |  |
| D | Allergies (seasonal, pets) |  |  |
| E | Daily weather changes |  |  |
| F | Having a cold |  |  |
| G | Humidity |  |  |
| H | Menstrual cycle |  |  |
| I | Alcohol |  |  |
| J | Smoking (being near others who smoke, or smoking) |  |  |
| K | Exercise |  |  |
| L | Changes in altitude |  |  |

- 1. Is there anything missing from this table? [Probe if needed:] If yes, tell me about those things.

1. Let’s move on to question **6**. What is this question asking you to do? [Interviewer: if participant gets it wrong, explain the intent of the question and then ask how we could make the intent clearer.]
   1. Let’s go through each row in question 6 using the think aloud technique again. Please choose a response for each row and tell me how you got to your answer. [Reminder: Record participant responses]

Which of the following symptoms do you experience outside of your episodes? (check one response per row)

|  |  | **Always or most of the time outside of episodes** | **Sometimes outside of episodes** | **I do not experience this symptom outside of episodes** | **I experience this symptom chronically** |
| --- | --- | --- | --- | --- | --- |
| A | Congestion |  |  |  |  |
| B | Headache |  |  |  |  |
| C | Sensitivity to sounds |  |  |  |  |
| E | Sensitivity to light |  |  |  |  |
| E | Watery or teary eyes |  |  |  |  |
| F | Blurry or hazy vision |  |  |  |  |
| G | Dizziness |  |  |  |  |
| H | Brain fog |  |  |  |  |
| I | Fatigue |  |  |  |  |
| J | Ear fullness |  |  |  |  |

- 1. Do you experience any symptoms between episodes that are missing from this table? [Probe if needed:] If yes, please tell me about those things.

1. Let’s move on to question **7**. What is this question asking you to do? [Interviewer: if participant gets it wrong, explain the intent of the question and then ask how we could make the intent clearer.]
   1. Let’s go through each row in question 7 with the think aloud technique. Please choose a response for each row and please tell me how you got to your answer. [Reminder: Record participant responses]

Do any of the following treatments help to address symptoms during your typical episodes? (check one response per row)

[Interviewer: Ask participant to clarify which specific medications helps them]

|  |  | **Always** | **Very often** | **Sometimes** | **Rarely** | **Never** | **I have not tried these** |
| --- | --- | --- | --- | --- | --- | --- | --- |
| A | Nasal steroid sprays (such as Flonase, Nasonex) |  |  |  |  |  |  |
| B | Antihistamines (such as Benadryl, Claritin, Zyrtec, Allegra) |  |  |  |  |  |  |
| C | Migraine medications (such as Imitrex/sumatriptan) |  |  |  |  |  |  |
| D | Antibiotics |  |  |  |  |  |  |
| E | Oral steroids |  |  |  |  |  |  |
| F | Caffeine |  |  |  |  |  |  |
| G | Over-the-counter pain reliever (such as Advil, Tylenol) |  |  |  |  |  |  |
| H | Decongestants (such as SudafedClaritin-D, Allegra-D, Afrin) |  |  |  |  |  |  |
| I | Injectable medications (such as Dupixant) |  |  |  |  |  |  |

1. Let’s look at questions **8 through 10**. Please read these questions to yourself.
   1. What response did you choose for each question? [Reminder: Record participant responses below] How did you get to your response?

| Q8 – family history of migraine |  |
| --- | --- |
| Q9 – unexplained headaches as a child |  |
| Q10 – motion sickness as a child |  |

1. Only a few more questions left! Let’s look at question **11**. Please read the question to yourself. Please tell me what response you choose.
   1. How did you decide this was the best response for you?
   2. [Interviewer: choose a response choice 1 up or 1 down from the response choice and ask:] Please describe how you would feel differently if you chose response choice x.


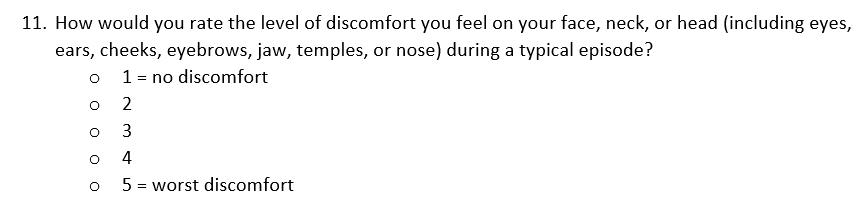


1. Looking at question **12** – please read the question to yourself. What response did you choose?
   1. How did you get to your response?
2. If there was a new medication available to improve your experiences with [xxx condition], what aspects of [xxx condition] are most important to you to be changed?
   1. [Probe:] Which symptoms?
   2. [Probe:]Which impacts? (social function, physical function, ability to work, ability to do hobbies)

Thank you very much for your help today.

Before we end the interview, I have a short demographics form for you to complete. The answers to these questions help us describe the people who participated in the study. We will report a summary of these data only.

Do you have any other questions? Thank you again. [we will mail you a check], as thanks for participating in this study.
